# Arithmetic value representation for hierarchical behavior composition

**DOI:** 10.1038/s41593-022-01211-5

**Published:** 2022-12-22

**Authors:** Hiroshi Makino

**Affiliations:** Lee Kong Chian School of Medicine, Nanyang Technological University, Singapore, Singapore

**Keywords:** Reward, Learning algorithms, Operant learning, Intelligence, Decision

## Abstract

The ability to compose new skills from a preacquired behavior repertoire is a hallmark of biological intelligence. Although artificial agents extract reusable skills from past experience and recombine them in a hierarchical manner, whether the brain similarly composes a novel behavior is largely unknown. In the present study, I show that deep reinforcement learning agents learn to solve a novel composite task by additively combining representations of prelearned action values of constituent subtasks. Learning efficacy in the composite task was further augmented by the introduction of stochasticity in behavior during pretraining. These theoretical predictions were empirically tested in mice, where subtask pretraining enhanced learning of the composite task. Cortex-wide, two-photon calcium imaging revealed analogous neural representations of combined action values, with improved learning when the behavior variability was amplified. Together, these results suggest that the brain composes a novel behavior with a simple arithmetic operation of preacquired action-value representations with stochastic policies.

## Abstract (web summary)

Using mice and artificial deep reinforcement learning agents trained in the same task, it is discovered that composition of a novel behavior entails a simple arithmetic operation on action values of constituent subtasks and their stochastic policies.

## Main

Humans and other animals can repurpose their preacquired behavior skills to new, unseen tasks. Such an aptitude can grow their behavior repertoire through combinatorial expansion^1–4^. Research in the artificial intelligence (AI) field of deep reinforcement learning (deep RL) posits that reuse of past experience can dramatically improve learning of tasks that can be broken down into simpler subproblems^5–9^. The linear arithmetic operation on prelearned action values (*Q*) derived from each subproblem leads to composition of a new nearly optimal policy, which can be transferred and further fine-tuned for a novel task^10,11^. However, it remains largely unknown whether the brain similarly creates a novel behavior.

In deep RL, policy entropy can be harnessed for agents to express a stochastic policy and learn multiple modes of nearly optimal behavior^12^. Maximum entropy policies endow agents with flexibility and robustness to perturbation. Furthermore, pretraining agents with maximum entropy policies enhance composability of a new behavior by providing better initialization to maintain the ability to explore in new settings^10,13^. In neuroscience, initially high behavior variability is similarly shown to be important for motor learning in humans and other animals^14,15^. Such a correspondence between artificial and biological systems prompts the question of whether there is algorithmic convergence to promote exploration for future learning.

As deep supervised and unsupervised learning have been pivotal to model neural activity in the visual system^16–19^, deep RL invites direct comparisons in representational learning underlying reward-based learning between the brain and the machine^20–22^. Inspired by the theoretical frameworks established in deep RL, in the present study I used cortex-wide, two-photon calcium imaging to empirically test whether these algorithmic features are leveraged in the mouse cortex while mice hierarchically solve a novel composite task. The results suggest that building blocks of stochastic policies acquired during pretraining can be combined to compose a nearly optimal policy for a downstream task with a minimum degree of fine-tuning.

## Results

### Hierarchical composition of a novel behavior in mice

**Fig. 1:**
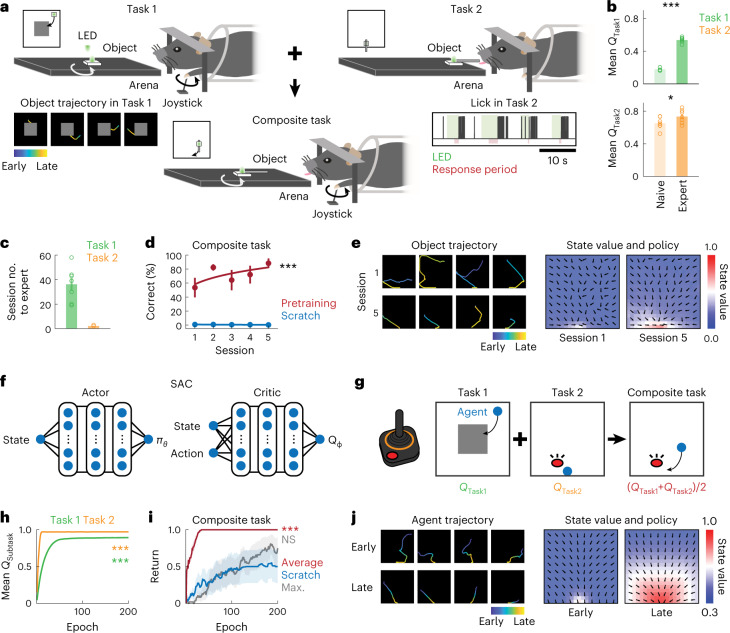
Hierarchical composition of a novel behavior in mice and deep RL agents. **a**, Schematic of the behavior paradigm for mice. Mice learned the two subtasks before the composite task. **b**, Increases in action values (*Q*) averaged over states and actions during subtask learning in mice (Task 1: ^***^P < 0.001, *n* = 6 and 7 mice for naive and expert, respectively; Task 2: ^*^P = 0.01, *n* = 7 mice for naive and expert, one-tailed bootstrap, mean ± s.e.m.). **c**, Number of sessions required to reach the expert stage for each subtask in mice (*n* = 7 mice for Tasks 1 and 2, mean ± s.e.m.). **d**, Learning curve for the composite task with and without pretraining in subtasks in mice (^***^P < 0.001 compared with the first session, *n* = 7 and 6 mice for pretraining and scratch, respectively, one-tailed bootstrap, mean ± s.e.m.). **e**, Example object trajectories (trial duration: session 1: 96.1 ± 27.5 s; session 5: 17.2 ± 9.3 s, mean ± s.e.m., 7 mice), state-value functions (color) and policies (vector field) in sessions 1 and 5 of the composite task in mice. **f**, Neural network architecture of deep RL agents trained with the SAC algorithm. *π*_*θ*_ is a policy, defined as an action probability given a state and parameterized by *θ.* *Q*_*φ*_ is an action value for a given state–action pair and parametrized by *φ*. **g**, Schematic of the behavior paradigm for deep RL agents. **h**, Increases in mean action values (*Q*) during subtask learning in deep RL agents (^***^P < 0.001 for Tasks 1 and 2, *n* = 6 agents, one-tailed bootstrap between naive and expert, mean ± s.e.m.). **i**, Learning curve for the composite task with average *Q*, maximum *Q* and without pretraining in subtasks in deep RL agents (average: ^***^P < 0.001 compared with scratch; maximum: NS, *P* = 0.89 compared with scratch, *n* = 6 agents, one-tailed bootstrap at the early stage with Bonferroni’s correction, mean ± s.e.m.). **j**, Same as **e** for deep RL agents. Early session: 20th epoch; late session: 200th epoch.

**Extended Data Fig. 1:**
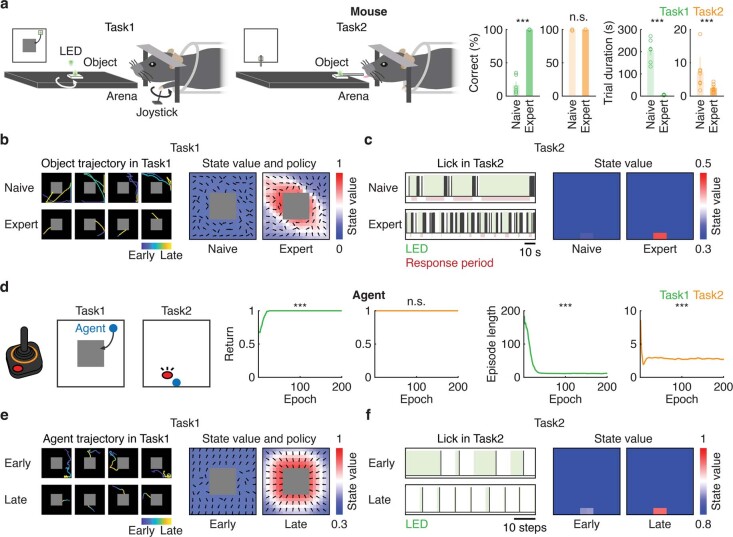
Behavior analysis of subtasks over learning in mice and deep RL agents. **a**, Left, schematic of subtasks. Right, subtask behavior analysis in mice (Task 1: ***P < 0.001, *n* = 7 and 6 mice for naive and expert respectively; Task 2: n.s., *P* = 0.33, ***P < 0.001, *n* = 7 mice for naive and expert, one-tailed bootstrap with Bonferroni correction, mean ± s.e.m.). Naive sessions in Task 1 were defined as the first few sessions when mice were directly introduced to the environment with the 4 × 4 cm^2^ reward zone and expert sessions were defined as those following completion of at least two consecutive sessions within 30 minutes. In Task 2, naive and expert sessions were defined as the first and fifth sessions, respectively. **b**, Example Task 1 trajectories, state-value functions, *V*(*s*), and policies, *π*, at different learning stages in mice (trial duration: naive: 204.7 ± 21.8 s; expert: 4.8 ± 0.6 s, mean ± s.e.m., *n* = 7 and 6 mice for naive and expert respectively). Note that the object movement was biased in top left and bottom right directions due to relative position of the joystick to the right forelimb. **c**, Example Task 2 lick events and state-value functions, *V*(*s*), at different learning stages in mice. Lick events required to dispense water but not related to water consumption became more restricted during the LED-on period over the course of learning (*P* = 0.006, *n* = 7 mice for naive and expert, one-tailed bootstrap). **d**, Subtask behavior analysis in deep RL agents (Task 1 and Task 2: ***P < 0.001, n.s., *P* = 1.0, *n* = 6 agents, one-tailed bootstrap between naive vs. expert with Bonferroni correction, mean ± s.e.m.). **e**, Same as **b** for deep RL agents. **f**, Same as **c** for deep RL agents.

I developed an object manipulation task in which head-restrained mice hierarchically combined two previously learned subtasks. In the first subtask (‘Task 1’), mice were trained to use a joystick to remotely move a light-emitting diode (LED)-attached object in an arena of 10 × 10 cm^2^ from a random location toward a reward zone in the center (4 × 4 cm^2^)^22^ (Fig. 1a and Extended Data Fig. 1a,b). Each trial was completed when the object successfully reached the reward zone (hit) or when 5 min had elapsed (miss). In the second subtask (‘Task 2’), mice were trained to lick a water spout placed on the stationary LED-attached object located in front of them (Fig. 1a and Extended Data Fig. 1a,c). Each trial ended when mice licked the water spout during a response period, which started 2 s after the LED onset (hit) or when 5 min had passed (miss). During Task 1, mice learned to manipulate the joystick to move (or not to move) the object (action) in each position of the arena (state). During Task 2, mice learned to associate the state of the LED-attached object (LED on/off status and its location) with licking (or no licking) action; over learning, their lick rate during the LED-on period became relatively higher than during the LED-off period (*P* = 0.008, *n* = 7 mice, one-tailed bootstrap). Subtask learning was evident from an increase in the correct rate or a decrease in the trial duration (Extended Data Fig. 1a). In each subtask, I measured the action-value function (*Q* function), an RL variable defined as the expected sum of future rewards when mice take a particular action *a* given a state *s* according to:

1 $$Q\left( {s,a} \right) = {\Bbb E}_\pi \left[ {R_{t + 1} + \gamma V\left( {s_{t + 1}} \right)} \right]$$

where ${\Bbb E}_\pi$ is an expectation under a policy *π*, *R*_*t*+1_ a reward at time *t* + 1 sampled every 10 ms, *γ* a discount factor (0.99) and *V*(*s*_*t*+1_) a state-value function defined as:

2 $$V\left( s \right) = {\Bbb E}_\pi \left[ {R_t + \gamma R_{t + 1} + \gamma ^2\,R_{t + 2} + \cdots + \gamma ^{T - t}R_T} \right]$$

where *T* is a trial end. As the reward was obtained only at the terminal state, *V*(*s*) can be simplified as^22^:

3 $$V\left( s \right) = {\Bbb E}_\pi \left[ {\gamma ^{T - t}} \right]$$

Thus, *Q* is a monotonic function of how fast either the object reaches the reward zone from each location (Task 1) or mice lick the water spout (Task 2) during the response period when the LED is on. Mean *Q* over states and actions increased during learning of these subtasks, with improvements in *V*(*s*) and *π* (Task 1: *P* < 0.001, *n* = 6 and 7 mice for naive and expert, respectively; Task 2: *P* = 0.01, *n* = 7 mice for naive and expert, one-tailed bootstrap; Fig. 1b and Extended Data Fig. 1b,c). These results demonstrate that mice learned to solve these subtasks more optimally.

**Extended Data Fig. 2:**
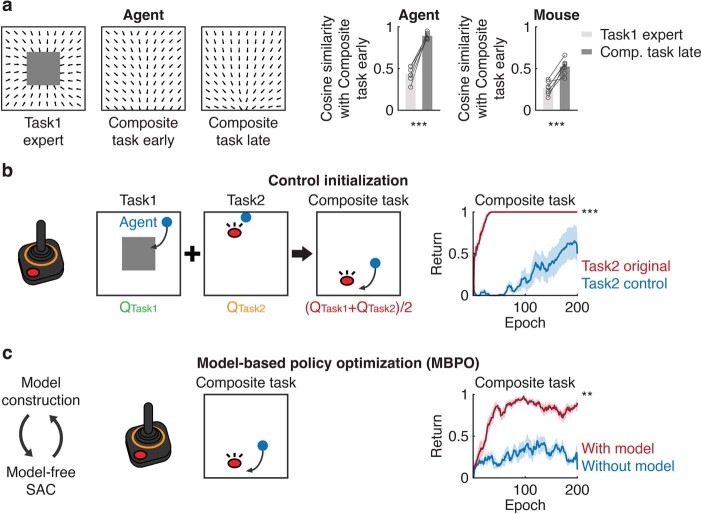
Further analysis of the composite task. **a**, Left, policies described as vector fields during each phase of the tasks in deep RL agents. Right, cosine similarities of the policies relative to the policy at the early phase of the composite task in deep RL agents and mice (***P < 0.001, *n* = 6 agents and *n* = 7 mice, one-tailed bootstrap, mean ± s.e.m.). **b**, Learning curve of deep RL agents with control initialization in the composite task (***P < 0.001, *n* = 6 agents, one-tailed bootstrap, mean ± s.e.m.). In the control initialization, Task 2 was modified so that the state of the agent was located at the top middle region of the arena. **c**, Learning curve of deep RL agents trained with and without model training in the model-based policy optimization (MBPO) algorithm in the composite task (**P = 0.006, *n* = 10 agents, one-tailed bootstrap, mean ± s.e.m.). MBPO constructs forward dynamics models and uses model-free SAC as a policy optimization algorithm under the learned model.

After mice became proficient at these subtasks, they were introduced to a new composite task where the water spout was attached to the object but was movable. In the composite task, mice combined preacquired knowledge in the two subtasks by moving the water spout (action) to a reachable location in the arena (state) while the LED was on (state) and lick the spout (action) to obtain a reward (Fig. 1a). I hypothesized that successful hierarchical composition of a new behavior was reflected by few-shot learning where only a few trials were necessary to achieve good performance. Although behavior training for the two subtasks took approximately 2–3 months (Fig. 1c), mice generally learned the composite task within one session (*P* < 0.001 compared with no pretraining, *n* = 6 and 7 mice for naive and expert, respectively, one-tailed bootstrap; Fig. 1d). Trajectory analysis revealed nearly optimal *V*(*s*) and *π* even in the first session (Fig. 1e). Notably, mice did not simply complete the two subtasks serially because the object was directed toward the bottom center of the arena during the early stage of the composite task (Fig. 1e and Extended Data Fig. 2a). These results demonstrate few-shot learning in mice through hierarchical combination of subtask policies.

### Hierarchical policy composition in deep RL agents

To build theoretical models to understand how the brain composes a novel behavior, artificial deep RL agents were trained in the same tasks with Soft Actor-Critic (SAC), a model-free actor-critic algorithm based on the maximum entropy RL framework^23,24^ (Fig. 1f,g). SAC is an off-policy algorithm that reuses past experiences to improve sample efficiency. SAC was selected because accumulating evidence suggests that animal learning may involve actor-critic-like mechanisms^21^ and its relevance to policy composition^10^. Although traditional actor-critic algorithms aim to maximize only the expected cumulative sum of future rewards, SAC additionally maximizes policy entropy according to the objective *J*(*π*):

4 $$J\left( \pi \right) = \mathop {\sum}\limits_{t = 0}^T {{\Bbb E}_{(s_t,a_t) \sim \rho _\pi }} \left[ {r\left( {s_t,a_t} \right) + \alpha {{{\mathcal{H}}}}\left( {\pi \left( { \cdot |s_t} \right)} \right)} \right]$$

where *ρ*_*π*_ is a state–action marginal of the trajectory distribution determined by *π,* *r*(*s*_*t*_,*a*_*t*_) a reward given a state *s*_*t*_ and action *a*_*t*_ at time *t*, *α* a temperature parameter to determine the relative contribution of the policy entropy term against the reward and ${{{\mathcal{H}}}}$ an entropy of *π*. *π*(·|*s*_*t*_) describes a probability of any action given a state *s*_*t*_. Intuitively, maximum entropy policies in SAC encourage exploration and capture multimodal solutions to the same problem by assigning a nonzero probability to all actions while sampling more promising avenues with a higher probability^13^. This property endows the agent with robustness to perturbation and flexibility. Importantly, theoretical analysis has demonstrated that (1) independently trained maximum entropy policies can be composed offline by adding their *Q* functions and (2) composability of a new policy depends on the entropy of the constituent subpolicies^10^. With a deep artificial neural network (ANN), the actor computes *π*, whereas the critic estimates the *Q* function. Following a previous study^23^, I constructed deep RL agents composed of actor and critic ANNs, each of which contained 3 hidden layers with 256 units (Fig. 1f). I confirmed that the agents trained for the subtasks improved task performance with gradual increases in their mean *Q* and *V*(*s*) and *π* being optimized (*P* < 0.001, *n* = 6 agents, one-tailed bootstrap; Fig. 1h and Extended Data Fig. 1d–f).

Research in deep RL established that individual *Q* functions obtained from each subtask can be averaged to derive a new composite *Q* (*Q*_Composite_) function to extract a new approximately optimal policy according to:

5 $$Q_{{\mathrm{Composite}}}^ \ast \left( {s,a} \right) \approx Q_\Sigma \left( {s,a} \right) = \frac{1}{{\left| C \right|}}\mathop {\sum}\limits_{i \in C} {Q_i^ \ast \left( {s,a} \right)}$$

where $Q_{{\mathrm{Composite}}}^ \ast$ is the true optimal *Q* function of the composite task, *Q*_Σ_ represents a newly derived composite *Q* function via averaging, *C* is defined as *C* ⊆ {1, …, *K*} of *K* tasks, |*C*| is the number of subtasks and $Q_i^ \ast$ the optimal *Q* function of the *i*th subtask^10,11^. This approximation has proven to be true when the constituent subpolicies agree on an action or are indifferent to each other’s actions^10^. The *Q*_Composite_ function acquired during pretraining can be transferred and further fine-tuned to be closer to the $Q_{{\mathrm{Composite}}}^ \ast$ function to solve a downstream task.

I tested whether such a simple arithmetic operation on subtask-derived *Q* functions (*Q*_Subtask_) enabled agents to solve the new composite task more efficiently (Fig. 1g). When introduced to the composite task, agents initialized with the combined *Q* function showed rapid learning compared with those learning from scratch (*P* < 0.001 compared with scratch, *n* = 6 agents, one-tailed bootstrap with Bonferroni’s correction; Fig. 1i). Agent’s trajectories and resulting *V*(*s*) and *π* were nearly optimal even at the early stage of training; the agents moved to the bottom center of the arena instead of serially completing the two subtasks (Fig. 1j and Extended Data Fig. 2a). By contrast, when the composite function was constructed by another method via computing the maximum of the two *Q*_Subtask_ functions, composite task learning remained slow (*P* = 0.89 compared with scratch, *n* = 6 agents, one-tailed bootstrap; Fig. 1i). Moreover, agents with control initialization by modifying Task 2 contained the same overall *Q* but failed to rapidly learn the composite task (*P* < 0.001, *n* = 6 agents, one-tailed bootstrap; Extended Data Fig. 2b). Notably, agents trained with the model-based policy optimization (MBPO) algorithm, which iteratively builds an ensemble of forward dynamic models and uses model-free SAC as a policy optimization algorithm under the learned model^25^, learned the composite task faster than those without the model (*P* = 0.006, *n* = 10 agents, one-tailed bootstrap; Extended Data Fig. 2c). This suggests that model construction can further augment the sample efficiency even without pretraining. These results reveal that averaging *Q*_*S*ubtask_ functions facilitated effective learning in the artificial agents.

### Emergence of neural representations of *Q*_Subtask_ functions

**Fig. 2:**
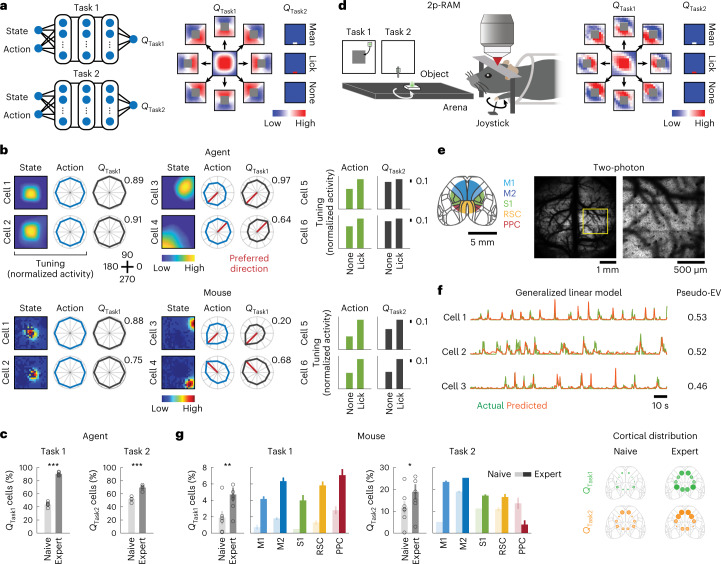
Emergence of *Q*_Subtask_ function representation during learning. **a**, Left, schematic of the ANNs in the deep RL agent. State and action scalar values are fed into the ANNs to output scalar values (*Q*_Task1_ and *Q*_Task2_). Right, *Q*_Subtask_ functions derived by computing *Q* in each state–action pair. The *Q*_Subtask_ functions were averaged across expert deep RL agents. The middle box in *Q*_Task1_ and the top box in *Q*_Task2_ indicate action-averaged *Q* functions in state spaces. For visualization, the spatial activation map for each action (movement in eight directions, lick and none) was obtained by subtracting the action-averaged *Q* function. The gray box denotes the reward zone. **b**, Space, direction and lick tuning of example neurons representing *Q* functions of Tasks 1 and 2 in expert deep RL agents (top) or mice (bottom). ‘State’ and ‘Action’ denote maximum-normalized activity. The number for each *Q* function describes the maximum *Q* value across directions. Two classes of *Q*_Task1_-representing neurons are displayed. **c**, Emergence of *Q*_Subtask_ function-encoding neurons during learning of Tasks 1 and 2 in deep RL agents (Task 1: ^***^P < 0.001; Task 2: ^***^P < 0.001, *n* = 6 agents, one-tailed bootstrap, mean ± s.e.m.). **d**, Left, schematic of the imaging experiment in the subtasks. Right, same as **a** for expert mice. **e**, Left, imaged cortical regions (M1, M2, S1, RSC and PPC). Right, example of two-photon calcium imaging. The right image is the boxed region in the left image. **f**, GLM for example neurons. Pseudo-EV denotes pseudo-explained variance. **g**, Left, same as **c** for mice (Task 1: ^**^P = 0.004, *n* = 6 and 7 mice for naive and expert, respectively; Task 2: ^*^P = 0.04, *n* = 7 mice for naive and expert, one-tailed bootstrap, mean ± s.e.m.). Neurons were further categorized based on cortical regions (fraction ± s.d. over 1,000 samples with replacement). Right, relative cortical distribution of *Q*_Task1_- and *Q*_Task2_-representing neurons across learning. Circle size and transparency indicate the relative fractions of neurons in the five cortical regions across the two learning stages.

**Extended Data Fig. 3:**
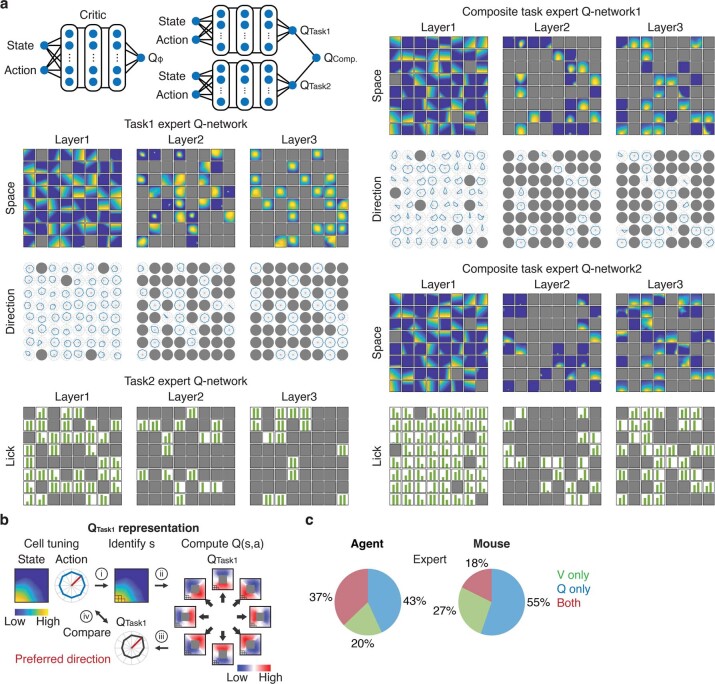
Neural representation in *Q*networks. **a**, Tuning properties for space, direction and lick in example units in each layer of *Q*networks for each task. **b**, Schematic of how *Q*_*Task*1_ representations in individual neurons were determined. After tuning properties of a given neuron were identified (i), relevant states, *s*, were determined based on space-related activity (ii). *Q*_*Task*1_ distribution over movement directions was then computed in these states (iii) and the resulting *Q*_*Task*1_ function was compared with the direction tuning of the neuron (iv). **c**, Fractions of each type of *Q*_*Task*1_-representing neurons in deep RL agents and mice.

To investigate neural mechanisms underlying the rapid composition of a novel behavior, I examined neural activity in hidden layers of the *Q* networks of the deep RL agents trained in each subtask (Fig. 2a and Extended Data Fig. 3a). In Task 1, I observed one type of neuron displaying high activity in the middle of the arena corresponding to high *V*_Task1_ (mean *Q*_Task1_ over actions), and the other type of neuron characterized by conjunctive space (state) and direction (action) tuning of the agent, where neuron’s directional tuning corresponded to the distribution of *Q*_Task1_ over actions in the state with high activity (Fig. 2b and Extended Data Fig. 3b). Intuitively, as the *Q*_Task1_ function is determined for each binned state and action pair, distributions over actions were compared between the unit activity and *Q* for the same states. Both classes of neurons were abundant (Extended Data Fig. 3c). In Task 2, a neuron was considered to be encoding the *Q*_Task2_ function when its lick tuning (activity during licking versus no licking) and *Q*_Task2_ (*Q* for licking versus no licking) were comodulated more than what would be expected by chance (Fig. 2b). Fractions of these *Q*_Subtask_ function-encoding neurons increased over learning in both subtasks (Task 1: *P* < 0.001; Task 2: *P* < 0.001, *n* = 6 agents, one-tailed bootstrap; Fig. 2c).

**Extended Data Fig. 4:**
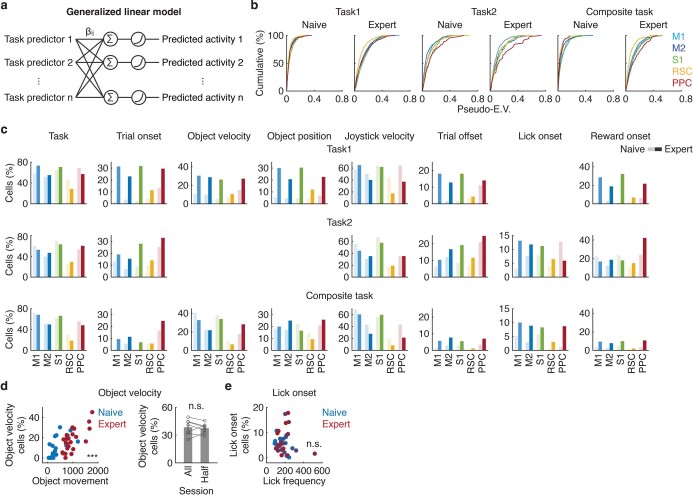
Generalized linear model (GLM) and representation of task variables. **a**, Schematic of GLM. Task predictors included trial onset and offset, object velocity, object position, joystick velocity, lick onset and reward onset. **b**, Pseudo-explained variance (EV) for each cortical region at the naive and expert stages of Task 1, Task 2 and the composite task. **c**, Fractions of task-variable-related cells in each cortical area. ‘Task’ indicates any of the task variables. **d**, Left, relationship between fractions of object velocity neurons and object movement (cm) in Task 1 (*R*^2^ = 0.50, ***P < 0.001 computed with Student’s *t* cumulative distribution function, two-tailed, *n* = 48 sessions, considering both the naive and expert stages). Right, fractions of object velocity neurons at the expert stage of Task 1 when all or the second half of the session was used (n.s., *P* = 0.41, *n* = 7 mice, one-tailed bootstrap, mean ± s.e.m.). These results indicate that the amount of the object movement *per se* was not related to the fractions of object velocity neurons; their co-modulation is a consequence of learning. **e**, Relationship between fractions of lick onset neurons and lick frequency (*R*^2^ = 0.01, n.s., *P* = 0.53 computed with Student’s *t* cumulative distribution function, two-tailed, *n* = 35 sessions, considering all learning stages).

**Extended Data Fig. 5:**
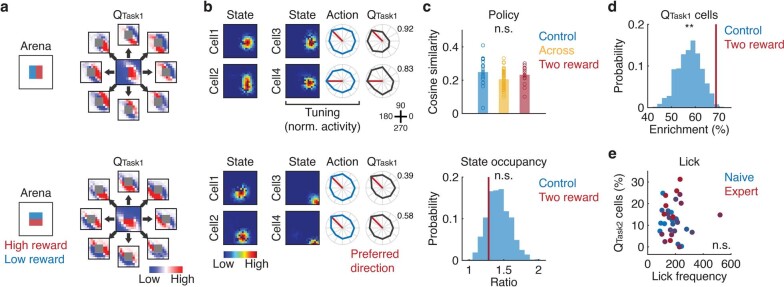
Neural representation of *Q*_*Subtask*_ functions is not due to actions *per se*. **a**, Environments with altered reward functions and resulting *Q*_*Task*1_ functions. The reward zone in Task 1 was split into two and a high or low reward was assigned. Same visualization as Fig. 2d. **b**, Tuning properties of example neurons showing shifts in space representations of *Q*_*Task*1_-representing neurons to the high reward side. **c**, The policy (top) and state occupancy (bottom) did not change between the original (control) and altered (two reward) reward functions (policy: n.s., *P* > 0.05 for all pair-wise comparisons, *n* = 21, 42, 15 animal-by-animal comparisons from the left to right, one-tailed bootstrap with Bonferroni correction, mean ± s.e.m.; state occupancy: n.s., *P* = 0.80, *n* = 7 and 6 mice for control and two reward, respectively, one-tailed bootstrap). For the policy, cosine similarities were computed either within or across the two environments. **d**, Quantification of the enrichment of *Q*_*Task*1_-representing neurons on the high reward side (**P = 0.006, *n* = 7 and 6 mice for control and two reward, respectively, one-tailed bootstrap, mean ± s.e.m.). **e**, Lick frequency was not predictive of the fractions of *Q*_*Task*2_-representing neurons (*R*^2^ = 0.00, n.s., *P* = 0.94 computed with Student’s *t* cumulative distribution function, two-tailed, *n* = 35 sessions, considering all learning stages).

To determine whether neural substrates of the *Q*_Subtask_ functions existed in the mouse brain, the activity of cortical excitatory neurons was imaged in transgenic mice (CaMKII-tTA × TRE-GCaMP6s) using a two-photon, random-access mesoscope (2p-RAM)^26^ (Fig. 2d,e). Calcium imaging with 2p-RAM records activity from thousands of neurons across distant cortical regions with cellular resolution. The imaging window included five cortical regions: primary motor cortex (M1), secondary motor cortex (M2), primary somatosensory cortex (S1), retrosplenial cortex (RSC) and posterior parietal cortex (PPC). Generalized linear models (GLMs) were built for individual neurons to extract their encoding properties for each task variable and their space and direction tuning was obtained (Task 1: 12,232 and 15,324 neurons; Task 2: 3,619 and 2,563 neurons for naive and expert, respectively; Fig. 2f and Extended Data Fig. 4a–c). Tuning properties analogous to those observed in the ANN emerged, such that the fractions of neurons encoding respective *Q*_Subtask_ functions increased over learning with distinct functional parcellation across cortical areas (Task 1: *P* = 0.004; *n* = 6 and 7 mice for naive and expert, respectively; Task 2: *P* = 0.04, *n* = 7 mice for naive and expert, one-tailed bootstrap; Fig. 2b,g). The observed fraction of neurons encoding the *Q*_Task1_ function at the expert stage (naive: 1.27%; expert: 5.11%) was above a chance level (naive: 0.60%; expert: 2.97%, *P* < 0.001 for both naive and expert, one-tailed permutation), computed by shuffling the indices of cells. Among neurons conjunctively tuned to space and direction, 29.2% of neurons were deemed to be representing the *Q*_Task1_ function (M1: 22.7%; M2: 34.5%; S1: 23.9%; RSC: 43.0%; PPC: 32.0%). The observed increase in the fractions of neurons encoding *Q*_Task1_ functions could not be explained by changes in the fractions of movement-related neurons (Extended Data Fig. 4d). Moreover, manipulation of the reward function confirmed that these neurons encoded the *Q*_Task1_ function but not the object movement itself (Extended Data Fig. 5a–d). In Task 2, the observed fraction of neurons encoding the *Q*_Task2_ function (naive: 11.8%; expert: 18.8%) was above a chance level (naive: 6.5%; expert: 9.2%, *P* < 0.001 for both naive and expert, one-tailed permutation), computed by shuffling of lick events. Furthermore, the fractions of neurons encoding the lick onset and *Q*_Task2_ function were not correlated with lick-event frequency (Extended Data Figs. 4e and 5e). At the expert stage, cortical distribution of *Q*_Subtask_ function-representing neurons for each subtask was distinct, where *Q*_Task1_-encoding neurons were more enriched in the PPC whereas *Q*_Task2_-encoding neurons were more abundant in M2 (Task 1 naive: M1 and M2: *P* < 0.01; M1 and PPC: *P* < 0.001; M2 and S1: *P* < 0.001; M2 and PPC: *P* < 0.01; S1 and RSC: *P* < 0.01; S1 and PPC: *P* < 0.001; RSC and PPC: *P* < 0.001; Task 1 expert: M1 and M2: *P* < 0.001; M1 and RSC: *P* < 0.01; M1 and PPC: *P* < 0.001; M2 and S1: *P* < 0.05; S1 and PPC: *P* < 0.001; Task 2 naive: M1 and M2: *P* < 0.001; M1 and S1: *P* < 0.001; M1 and RSC: *P* < 0.001; M1 and PPC: *P* < 0.01; M2 and S1: *P* < 0.001; M2 and RSC: *P* < 0.001; Task 2 expert: M1 and M2: *P* < 0.001; M1 and S1: *P* < 0.001; M1 and RSC: *P* < 0.001; M1 and PPC: *P* < 0.001; M2 and S1: *P* < 0.001; M2 and RSC: *P* < 0.001; M2 and PPC: *P* < 0.001; S1 and PPC: *P* < 0.001; RSC and PPC: *P* < 0.001; all the other comparisons: *P* > 0.05, two-tailed bootstrap with false discovery rate (FDR); Fig. 2g). These results are corroborated by our previous study demonstrating that the PPC is critical for the object manipulation task^22^, with others demonstrating the importance of anterior lateral motor cortex (a subregion of M2) for licking behavior^27^. Thus, the mouse cortex learned to represent the *Q*_Subtask_ functions in functionally segregated networks.

### Transfer of learned representations of *Q*_Subtask_ functions

**Fig. 3:**
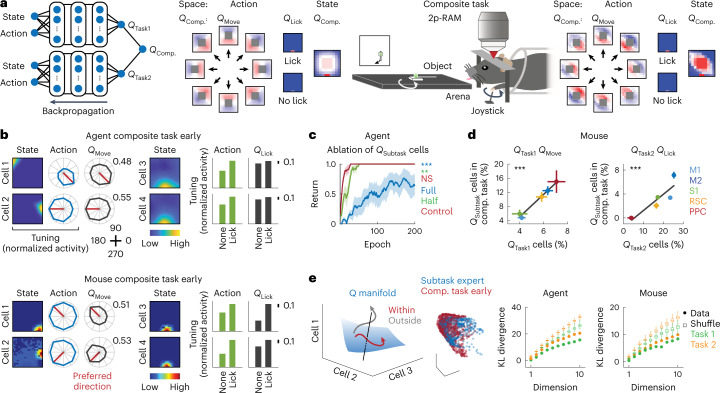
Transfer of *Q*_Subtask_ representation to the composite task. **a**, Left, schematic of the composite ANN architecture and the *Q*_Composite_ function in action and state spaces in deep RL agents in the early learning phase of the composite task. Output scalar values (*Q*_Task1_ and *Q*_Task2_) are averaged to derive a scalar value (*Q*_Composite_ (*Q*_Comp_.)). Right, same as left for mice with schematic of the imaging experiment. It is the same visualization as Fig. 2a,d. **b**, *Q*_Subtask_ representations in the early phase of the composite task in deep RL agents (top) and mice (bottom). It is the same visualization as Fig. 2b. **c**, Learning curve in the composite task with ablation of *Q*_Task1_- and *Q*_Task2_-representing neurons in deep RL agents. Full ablation: 11.3 ± 0.3% (mean ± s.e.m.) for movement and 14.0 ± 0.3% for lick of all neurons ablated, ^***^P < 0.001 compared with control ablation; 50% ablation: 5.8 ± 0.1% for movement and 7.1 ± 0.2% for lick of all neurons ablated, **P = 0.006 compared with control ablation; control ablation: same number as the full ablation ablated, *P* = 0.83 compared with no ablation, *n* = 6 agents, one-tailed bootstrap with Bonferroni’s correction (mean ± s.e.m.). **d**, Persistent cortical distribution of *Q*_Subtask_-related movement (left) and lick (right) representations across the subtasks and composite (comp.) task in mice (Task 1: *R*^2^ = 0.98, ^***^P < 0.001; Task 2: *R*^2^ = 0.76, ^***^P < 0.001, one-tailed bootstrap for positive correlations, fraction ± s.d. >1,000 samples with replacement). Direct comparisons in the absolute fractions of *Q*_Subtask_-related neurons across tasks are complicated due to different task predictors used in GLMs. **e**, Left, schematic of the *Q* manifold of population neural activity representing a *Q* function. Middle, *Q* manifold in an example of a deep RL agent embedded in the same principal component space. The dots correspond to activity at randomly sampled timepoints. Right, manifold overlaps. The dots indicate the mean KL divergence. The edges of the whiskers are maximum and minimum and the edges of the boxes are 75% and 25% of 1,000 shuffled mean KL divergence (*P* < 0.001 for all comparisons, *n* = 7 mice and *n* = 6 agents, one-tailed permutation).

**Extended Data Fig. 6:**
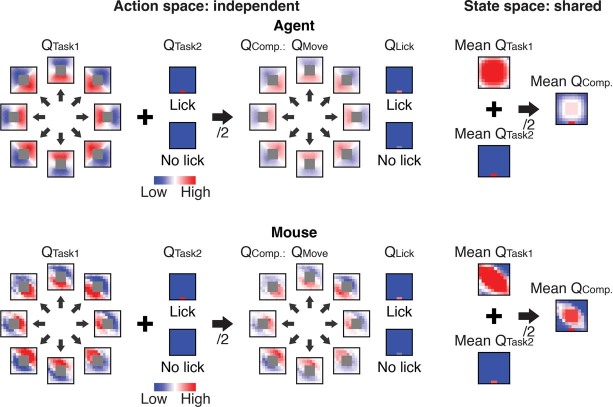
Representation models for the hierarchical composition of a new *Q*_*Composite*_ function. Left, since the action spaces between Task 1 and Task 2 are independent, averaging the two *Q*_*Subtask*_ functions yields a similar *Q* function (*Q*_*Comp*._). Right, by contrast, since the state spaces are shared between Task 1 and Task 2, the averaging results in a mixed value function with two hot spots located in the center and bottom middle regions. Same visualization as Fig. 2a,d.

**Extended Data Fig. 7:**
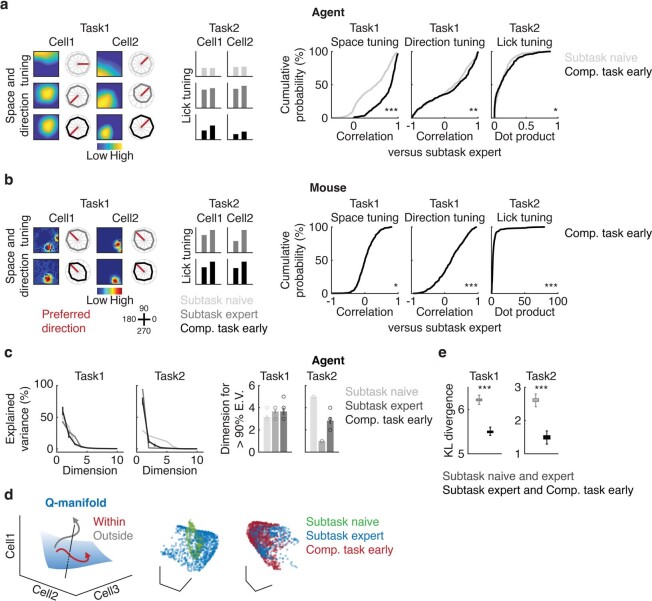
Reuse of learned neural representation in the composite task. **a**, Neural representations in deep RL agents. Left, space, direction and lick tuning of example neurons at the subtask naive, subtask expert and composite task early stages. Right, quantification of representation similarity. Space tuning: ***P < 0.001; direction tuning: **P = 0.005, *n* = 478 and 403 neurons for Task 1 naive and composite task early compared to Task 1 expert, respectively; lick tuning: *P = 0.02, *n* = 427 and 413 neurons for Task 2 naive and composite task early compared to Task 2 expert, respectively, two-tailed KS test with Bonferroni correction). **b**, Same as **a** for mice. Note that the same neurons were imaged across the subtask expert and composite task early stages only (no subtask naive stage). Space tuning: *P = 0.03, *n* = 422 neurons; direction tuning: ***P < 0.001, *n* = 634 neurons; lick tuning: ***P < 0.001, *n* = 213 neurons, one-tailed permutation with Bonferroni correction). **c**, Left, explained variance (EV) derived from principal component analysis (PCA) as a function of dimensions across different learning stages of deep RL agents (*n* = 6 agents for Task 1 and Task 2, mean ± s.e.m.). Right, number of dimensions required for EV of >90% across different learning stages in deep RL agents (*n* = 6 agents for Task 1 and Task 2, mean ± s.e.m.). **d**, Schematic of the *Q*manifold and example population activity of deep RL agents in the PC space. Note that the overlap between the subtask naive versus subtask expert stages is smaller than the subtask expert versus composite task early stages. **e**, Manifold overlaps measured by KL divergence of the population activity in the PC space in deep RL agents (Task 1 and Task 2: ***P < 0.001, *n* = 6 agents, one-tailed bootstrap). Number of dimensions considered: 3 for Task 1 and 1 for Task 2 based on **c**. The edges of the whiskers are maximum and minimum and the edges of the boxes are 75% and 25% of 1000 randomly sampled mean KL divergence.

In the deep RL agents, few-shot learning in the composite task was attained by constructing the *Q*_Composite_ function via averaging the two *Q*_Subtask_ functions (Fig. 3a). I examined the consequence of such an arithmetic operation on neural representations of the *Q* function in the ANN of the deep RL agents at the early learning stage of the composite task (Supplementary Video 1). During training of the ANN, backpropagation of the error signal derived from the new *Q*_Composite_ function modulates neural activity across the whole network (Fig. 3a). Two predictions were made in the present study: first, as the action spaces in Task 1 (movement) and Task 2 (lick) were independent, averaging of *Q*_Task1_(*s*,*a*) and *Q*_Task2_(*s*,*a*) yields representations similar to those of individual *Q*_Subtask_(*s*,*a*), albeit the values are halved (Extended Data Fig. 6). The *Q* functions related to the object movement and licking were referred to as *Q*_Move_ and *Q*_Lick_, respectively, regardless of whether the task was a subtask or composite task, whereas *Q*_Subtask_ denoted the *Q* function for the object movement in Task 1 or licking for Task 2. Second, as the state spaces were shared between Tasks 1 and 2 (position of the agent), state representations derived via averaging reveal a mixed *Q*_Subtask_ function (Extended Data Fig. 6). Overall, response profiles in individual neurons were more similar during the transition from the subtasks to the composite task than during learning of the individual subtasks (Extended Data Fig. 7a). Consistent with the first prediction, representations of the *Q*_Subtask_ functions were retained in the composite task (Fig. 3b). The importance of these representations was evident as ablation of *Q*_Subtask_-encoding neurons in the ANN decelerated learning of the composite task in the deep RL agents (full ablation: *P* < 0.001; 50% ablation: *P* = 0.006, *n* = 6 agents, one-tailed bootstrap with Bonferroni’s correction), whereas control ablation retained few-shot learning (*P* = 0.83 compared with no ablation, *n* = 6 agents, one-tailed bootstrap, Fig. 3c). These results establish that representations of *Q*_Subtask_ functions in the ANN were reused in the composite task at the level of single neurons.

I reasoned that, if the few-shot learning of the composite task in mice was mediated by a similar mechanism, representations in cortical neurons should resemble those detected in the deep RL agents. Indeed, tuning properties of single neurons remained comparable between the subtasks and composite task (3,364 and 4,873 neurons analyzed in the composite task for the early and late sessions, respectively; Fig. 3b and Extended Data Fig. 7b). The observed fraction of neurons encoding the *Q*_Task1_ function at the early stage of the composite task (8.3%) was above a chance level (5.3%, *P* < 0.001, one-tailed permutation). Among neurons conjunctively tuned to space and direction, 39.9% of neurons were deemed to represent the *Q*_Task1_ function in the composite task (M1: 30.1%; M2: 46.5%; S1: 25.8%; RSC: 52.0%; PPC: 60.0%). Moreover, cortical distribution of the *Q*_Subtask_-function representations was stable such that functionally specialized circuits persisted across the subtasks and composite task (Fig. 3d).

Why was learning of the composite task more sample efficient in the deep RL agents and mice? It has been proposed that learning is constrained by the intrinsic covariance structure of neural population activity^28^: learning becomes harder when the covariance needs large restructuring. I hypothesized that the rapid acquisition of a new behavior was possible due to reuse of the preacquired patterns of population neural activity. Population activity can be described as a point in high-dimensional space where each axis corresponds to the activity of a single neuron. Comodulation of activity of a population of neurons comprises a low-dimensional subspace, known as the intrinsic manifold^28^. With Kullback-Leibler (KL) divergence estimation^29^, divergence of the intrinsic manifolds was measured between the expert stage of the subtasks and the early stage of the composite task. This analysis revealed that similar intrinsic manifolds were shared across tasks in both the deep RL agents and the mice (Fig. 3e and Extended Data Fig. 7c–e). By contrast, subtask learning in the deep RL agents considerably changed intrinsic manifolds, indicating that there was large reorganization of weights in the ANN (Extended Data Fig. 7c–e). This observation also provides a potential explanation of why learning of the subtasks was slow in mice (Fig. 1c). Together, these results demonstrate that the deep RL agents and mice transferred geometric representations of learned task variables to efficiently solve the novel task. In the case of the deep RL agents, as representation learning acts as a bottleneck^30^, reuse of learned representations of the *Q*_Subtask_ functions improves sample efficiency in the downstream task.

### Hierarchical composition of *Q*_Composite_ representations

**Fig. 4:**
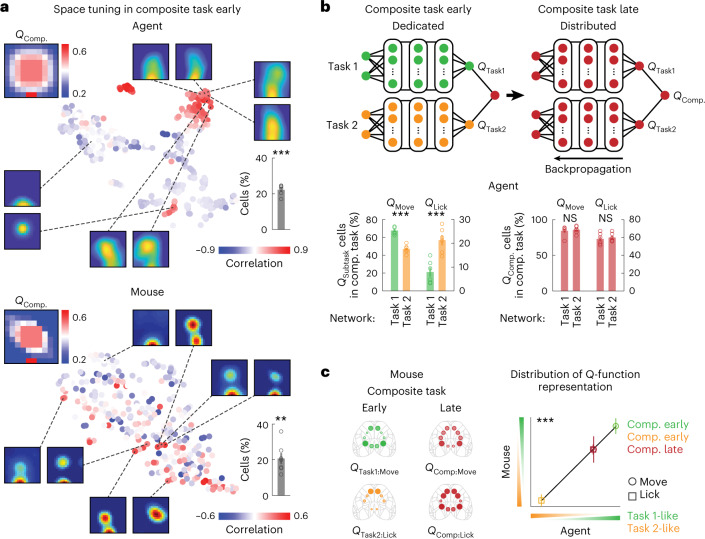
Arithmetic operation on *Q*_Subtask_ representation in the composite task. **a**, Space tuning of 300 randomly selected neurons in the tSNE coordinate for deep RL agents and mice. The color indicates Pearson’s correlation coefficient between space tuning and the spatial map of the *Q*_Composite_ function. Note the two locations (center and bottom middle) corresponding to the high value states derived from the two subtasks (agent: ^***^P < 0.001, *n* = 6 agents, mouse: ^**^P = 0.006, *n* = 7 mice, one-tailed bootstrap compared with a chance level, mean ± s.e.m.). **b**, Top, schematic of the composite ANN architecture of the deep RL agent and changes in representation during the composite task learning. Representations of movement-related and lick-related *Q* in both subnetworks become more distributed due to backpropagation of the common error signal. Bottom, distribution of movement- and lick-related *Q*_Subtask_- and *Q*_Composite_-representing neurons in each subnetwork in deep RL agents across learning (^***^P < 0.001, nonsignificant (NS): *P* = 0.66 and 0.40 from the left to right, *n* = 6 agents, one-tailed bootstrap, mean ± s.e.m.). *Q*_*M*ove_ and *Q*_Lick_ are action values for the object movement and licking, respectively, derived from the subtask or the composite task. **c**, Left, cortical distribution of movement- and lick-related *Q*_Subtask_- and *Q*_Composite_-representing neurons. The circle size and transparency indicate the relative fractions of neurons in the five cortical regions across the two learning stages. Right, distribution of *Q* representations in deep RL agents constructed by arithmetic operation of the *Q*_Subtask_ functions predicts the cortical distribution of *Q* representations in mice (*R*^2^ = 1.0, ^***^P < 0.001, one-tailed bootstrap for positive correlations, error bars: s.d. of 1,000 samples with replacement). The two axes denote how dedicated *Q* representations are in each subnetwork for deep RL agents and in cortical distributions for mice. For example, the green open circle indicates dedicated Q_Move_ representations at the early phase of the composite task in the Task 1 subnetwork for deep RL agents, whereas cortical distributions of *Q*_Move_ representations were similar between the early phase of the composite task and the expert stage of Task 1 for mice.

**Extended Data Fig. 8:**
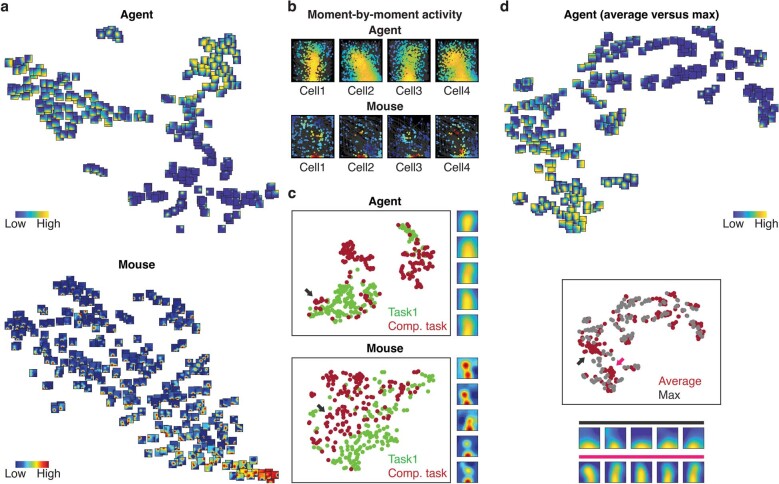
Further analysis of the hierarchical composition a new *Q*_*Composite*_ function. **a**, Space tuning of individual neurons shown in Fig. 4a for deep RL agents and mice. **b**, Moment-by-moment activity of example neurons encoding the *Q*_*Composite*_ function. The activity of the mouse neurons was derived from GLM after marginalizing out other task predictors than the object position. **c**, Spatial representations of randomly selected 300 neurons (150 from Task 1 and 150 from the composite task) in the same tSNE coordinate are distinct between Task 1 and the composite task in deep RL agents and mice. The arrows indicate regions containing the mixed *Q* representations shown on the right. **d**, Spatial representations of randomly selected 300 neurons in the same tSNE coordinate in deep RL agents trained with the *Q*_*Composite*_ function derived from the average and maximum of the *Q*_*Subtask*_ functions at the early stage of the composite task (150 from average and 150 from maximum). The magenta and gray arrows indicate clusters of neurons whose space tuning was unique to the *Q*_*Composite*_ function derived from the averaging operation. Note that such a subtle difference led to distinct learning efficiency in the composite task (Fig. 1i), indicating that these mixed *Q* representations of the subtasks (high activation in the center and bottom middle regions of the arena) were important for the hierarchical composition of a new policy.

It has been demonstrated so far that reuse/transfer of *Q*_Subtask_ representations in the deep RL agents facilitates composite task learning. In mice, similar *Q* representations were observed between the subtasks and composite task. However, construction of the new *Q*_Composite_ function and its fine-tuning were critical because mere coexistence of independent *Q* representations of the two subtasks was not sufficient to fully solve the composite task (first training epoch in ‘average’ on Fig. 1i). Existence of the individual *Q*_Subtask_ representations in mice supports, but does not prove, a new *Q*_Composite_ function being constructed through the averaging operation. To address this, I tested the second prediction that the state representation derived from averaging results in a mixture of *Q*_Subtask_ functions in single neurons (Extended Data Fig. 6). Based on Pearson’s correlation coefficients between the state representation of the *Q*_Composite_ function and space tuning of individual units, I confirmed the second prediction to be true even at the early stage of learning in the composite task; spatial activation of neurons was confined to two locations corresponding to the states with high expected values of the *Q* in the two subtasks (Fig. 4a and Extended Data Fig. 8a). The resulting neural representations were due to moment-by-moment activation of each neuron at the corresponding states (Extended Data Fig. 8b). The observed fraction (22.2%) was higher than what would be expected by chance assuming that space tuning was uniformly distributed (11.1%, *P* < 0.001, one-tailed bootstrap). These activation patterns were rarely observed in the subtasks (Fig. 2b and Extended Data Fig. 8c). In addition, when the *Q*_Composite_ function was derived from computing the maximum of the two *Q*_Subtask_ functions, differences in representations from the averaging operation were subtle, except that these mixed *Q*_Subtask_ representations were absent (Extended Data Fig. 8d). As such a small difference led to distinct learning efficiency in the composite task (Fig. 1i), the mixed representations were likely to be critical in the deep RL agents.

In mice, more direct evidence for the additive *Q*_Composite_ construction is, therefore, to reveal similar multiplexed representations of *Q*_Task1_ and *Q*_Task2_ in single cortical neurons. Neurons in the mouse cortex were indeed spatially tuned to the two high value states derived from the two subtasks (Fig. 4a and Extended Data Figs. 6 and 8a). The observed fraction (20.7%) was higher than what would be expected by chance assuming that space tuning was uniformly distributed (16.1%, *P* = 0.006, one-tailed bootstrap). As in the case of the ANN, the mixed representations were due to moment-by-moment activation of each neuron, do not reflect co-occurrence of the object movement and licking actions, and were not observed in the subtasks (Extended Data Fig. 8b,c). Importantly, these tuning properties did not reflect a reward as such because there was no reward associated with the center of the arena in the composite task. Furthermore, the observed representations suggest that the brain’s efficient learning in the composite task was not simply attained by construction of dynamic models (Extended Data Fig. 2c), but the combination of prelearned *Q* functions is important to derive a new policy.

**Extended Data Fig. 9:**
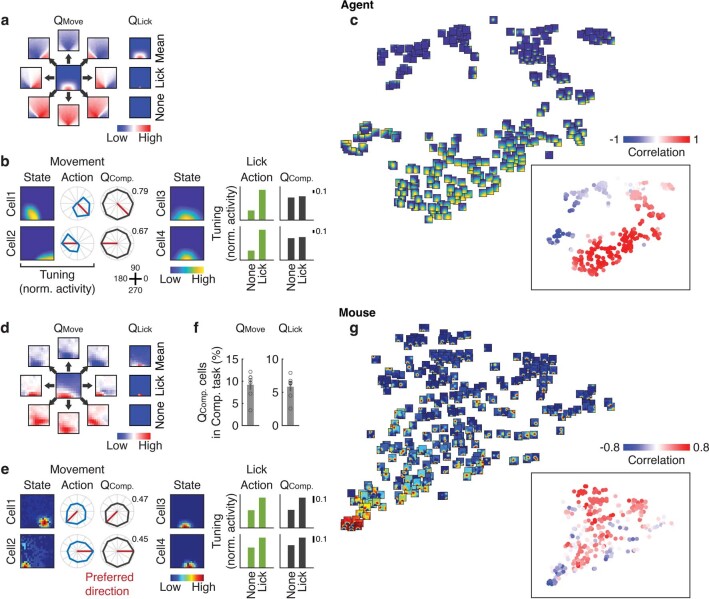
Neural representation of the *Q*_*Composite*_ function at the late learning stage of the composite task. **a**, *Q*_*Composite*_ function in deep RL agents at the late learning stage of the composite task. Same visualization as Fig. 2a. **b**, Example neural representations of the *Q*_*Composite*_ function in deep RL agents. Same visualization as Fig. 2b. **c**, Space tuning of randomly selected 300 neurons in the tSNE coordinate in deep RL agents. Correlation denotes Pearson correlation coefficient between space tuning of individual neurons and the mean *Q*_*Composite*_ function over actions. **d**, Same as **a** for mice. **e**, Same as **b** for mice. **f**, Fractions of movement- and lick-related *Q*_*Composite*_-representing neurons at the late stage of the composite task in mice (*n* = 7 mice, mean ± s.e.m.). Corresponding plots for deep RL agents are shown in Fig. 4b. **g**, Same as **c** for mice.

The rapid learning in the composite task in the deep RL agents and mice was followed by gradual refinement of the policy during the fine-tuning phase (Fig. 1e,j). To seek additional evidence for the composition of the new *Q*_Composite_ function in mice, I next studied how neural representations were shaped during this phase of learning. As the deep RL agents learned the composite task (Supplementary Video 2), there was a transition in ANN activity from dedicated to distributed representations: neural representations of *Q* for movement and lick in the two subnetworks, which were computed in the same manner as *Q*_Subtask_ representations, were initially segregated but gradually became mixed on training (Fig. 4b and Extended Data Fig. 9a–c). I reasoned that if the mouse cortex computed the *Q*_Composite_ function by averaging the two *Q*_Subtask_ functions, there should be similar redistribution of neural representations of the *Q* function across the cortical regions. In support of this, although *Q*_Subtask_ representations were initially segregated in dedicated circuits, *Q*_Composite_ representations became widely distributed across different cortical regions after the composite task learning (Figs. 2g and 4c and Extended Data Fig. 9d–g). Remarkably, redistribution of the agent’s *Q* representations in the subnetworks predicted that observed in the mouse cortex (*R*^2^ = 1.0, *P* < 0.001, one-tailed bootstrap for positive correlations; Fig. 4c). These similarities lend further support to the notion that mice used a simple arithmetic operation to derive a new *Q*_Composite_ function to solve the composite task.

### Maximum entropy policy for efficient behavior composition

**Fig. 5:**
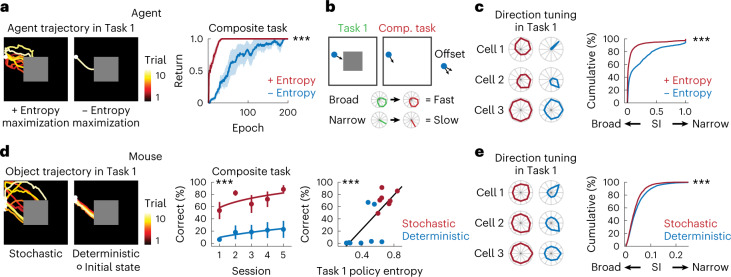
Maximum entropy policies improve learning of the composite task. **a**, Left, example trajectories of deep RL agents in Task 1 with and without policy entropy maximization. Note that the trajectories overlap in the right panel. Right, learning curve in the composite task with and without policy entropy maximization (^***^P < 0.001, *n* = 6 agents, one-tailed bootstrap at the early stage, mean ± s.e.m.). **b**, Schematic showing that broader direction tuning confers rapid learning in the composite task because minimal fine-tuning is required. **c**, Example direction tuning of the same neurons in deep RL agents in Task 1 with and without policy entropy maximization, measured by the SI defined as 1 − Circular variance (^***^P < 0.001, *n* = 2,340 and 2,313 cells with and without policy entropy maximization, respectively, one-tailed KS test). **d**, Left, example trajectories of the object moved by mice in Task 1 with stochastic and deterministic policies. Middle, learning curve in the composite task with stochastic and deterministic policies (^***^P < 0.001 compared with the first session, *n* = 7 and 8 mice for stochastic and deterministic policy, respectively, one-tailed bootstrap, mean ± s.e.m.). Right, correlation between Task 1 policy entropy and the session-averaged correct rate in the composite task (*R*^2^ = 0.57, ^***^P < 0.001 computed with Student’s *t* cumulative distribution function, one tailed, *n* = 15 mice). **e**, Example of direction tuning of neurons in the mouse cortex in Task 1 with stochastic and deterministic policies (^***^P < 0.001, *n* = 3,251 and 2,972 cells for stochastic and deterministic policy, respectively, one-tailed KS test).

**Extended Data Fig. 10:**
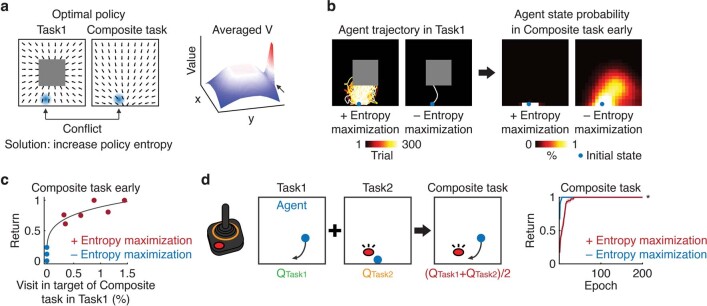
Maximum entropy policies improve composability of a new policy. **a**, Left, schematic illustrating the conflict in policies between Task 1 and the composite task. Right, *V*(*s*) in the composite task averaged across deep RL agents. The arrow indicates location of the conflict. **b**, Maximum entropy policies for Task 1 increase the probability of deep RL agents to visit the reward zone in the composite task. The higher visitation of the reward zone of the composite task in Task 1 increases the state occupancy in its vicinity during the early phase of the composite task. **c**, Relationship between the visitation of the reward zone of the composite task in Task 1 and return during the early stage of the composite task (*R*^2^ = 0.59 for ‘+ entropy maximization’ fit with a single-term power series model, *n* = 6 agents for ‘+ entropy maximization’ and ‘– entropy maximization’). **d**, Maximum entropy policies may become detrimental when the target regions are identical between Task 1 and the composite task. In this case, deterministic policies in Task 1 lead to more rapid learning, albeit at the cost of flexibility (*P = 0.01, *n* = 6 agents, one-tailed bootstrap, mean ± s.e.m.).

Finally, I examined whether a stochastic policy was critical for the rapid composition of the new behavior, a question related to the second term of the objective in the SAC algorithm. In the deep RL agents, behavior performance in the composite task depended on the policy entropy, such that learning was accelerated when the agent’s policy in Task 1 became stochastic: when the entropy maximization term was removed from the RL objective of the SAC algorithm (*α* = 0 in equation (4)), pretraining with the subtasks failed to improve learning (*P* < 0.001, *n* = 6 agents, one-tailed bootstrap; Fig. 5a). Additional analysis demonstrated that, even though there was a conflict in the optimal policies between Task 1 and the composite task, entropy in subpolicies promoted exploration such that it enhanced the probability of visiting the reward zone in the composite task; the visitation of the reward zone predicted whether the agents were successful in solving the composite task (Extended Data Fig. 10a–c). Notably, the maximum entropy policy can be detrimental under the condition that the reward zone between Task 1 and the composite task was identical (*P* = 0.01, *n* = 6 agents, one-tailed bootstrap; Extended Data Fig. 10d). Furthermore, successful composition of a new policy entailed a substantial overlap in high *Q* states between the subtasks and composite task (Extended Data Fig. 2b).

I hypothesized that the deep RL agents’ few-shot learning of the composite task was due to representations of broadly distributed *Q* functions over different actions, which enable multimodal solutions^12^. Depending on the state, the optimal action of the agent could be slightly different between Task 1 and the composite task, and the agents were required to fine-tune their policy accordingly. Broad representations of *Q* functions can flexibly adapt to such a slight offset in the optimal policy (Fig. 5b). However, if representations of *Q* are specific to certain directions, the agents need to unlearn old *Q* functions and relearn new ones. When the agents were trained with the entropy maximization term, the direction tuning of individual neurons was broader (*P* < 0.001, *n* = 2,340 and 2,313 cells with and without entropy maximization, respectively, one-tailed Kolmogorov–Smirnov (KS) test, Fig. 5c).

I next tested whether policy stochasticity was similarly important in mice by modifying Task 1 to encourage mice to employ a more deterministic policy (Fig. 5d). As observed in the deep RL agents, learning in the composite task was slower when the trajectories in Task 1 were not highly variable (*P* < 0.001, *n* = 7 and 8 mice for stochastic and deterministic policy, respectively, one-tailed bootstrap; Fig. 5d). Individually, the variability in the Task 1 policy entropy predicted the variability of task performance in the composite task (*R*^2^ = 0.57, *P* < 0.001, *n* = 15 mice, one-tailed for correlation; Fig. 5d). Neural representations of the object movement direction were broader when the policy was more stochastic (*P* < 0.001, *n* = 3,251 and 2,972 cells for stochastic and deterministic policy, respectively, one-tailed KS test; Fig. 5e). Thus, policy entropy conferred flexible composition of a new behavior policy by broadly representing *Q* functions over different actions.

## Discussion

Although research in AI aims to express unique properties of biological intelligence in machines^3^, various algorithms have been developed to provide explanatory views of their biologically plausible mechanisms. This has, in turn, created new opportunities for neuroscience to empirically validate theoretical models of how biological intelligence is implemented^31–33^. It has been postulated that new ideas and behavior skills are rapidly developed through a combination of their primitives, the notion related to compositionality^9,34^. Such properties, however, have rarely been scrutinized in rodent system neuroscience, in which recordings of single neurons across multiple brain areas are commonplace. Deep RL, on the other hand, reveals neural representations of RL variables in single neurons in the ANN. Exploiting advantages of both systems, I generated deep RL-derived models on how concerted actions of individual neurons in the brain lead to new policy composition. A simple linear operation of averaging, but not computing the maximum of, the *Q*_Subtask_ functions in the deep RL agents was critical to increase sample efficiency in the composite task. A striking resemblance between the deep RL agents and mice in their behavior and *Q* representations implies that animals employ a similar arithmetic code to expand their behavior repertoire.

Brain regions such as the basal ganglia, in particular dorsal and ventral subdivisions of the striatum, are often associated with the actor-critic model^35,36^. The present results indicate additional involvement of cortical neurons in representing values of relevant actions according to the functional parcellation, which is consistent with the proposed role of corticostriatal pathways^35,37^. This view is corroborated by a recent human study demonstrating that activity in the PPC resembles activation patterns in the deep *Q* network^38^. The current finding, however, does not exclude the possibility that other forms of learning not involving a reward, or other brain regions not examined in the present study, contribute to the composition of a novel behavior. For instance, the brain may construct predictive models of the environment to further improve learning via simulations. In the present study, I demonstrate that construction of dynamic models and model-free policy optimization together augments sample efficiency in the deep RL agents (Extended Data Fig. 2c). Whether the brain constructs dynamic models, in addition to the transfer of the combined *Q* function described in the present study, warrants further investigation.

Variability in the sensorimotor system can be harnessed to augment learning in humans and other animals^14,15^. In deep RL, the SAC algorithm maximizes the policy entropy to attain multiple solutions to the same problem, which has been theoretically shown to improve composability of a new policy: stochastic and deterministic subpolicies are associated with high and low composability, respectively^10^. In the context of the present study, deterministic policies in the subtasks during pretraining lead to loss of exploration when they are transferred to the composite task, leading to very slow adaptation of the agents. Such a common mechanism between the biological and artificial systems uncovers an essential role of variability in behavior composition. In particular, the comparative analysis provides a formal account of why variability facilitates learning in the biological system. High variability in a policy allows exploration of relevant *Q* spaces, which was evident in broadly tuned, action-related neural activity in both systems. I acknowledge that the manipulation to encourage mice to employ a deterministic policy might have deprived them of additional statistical features of the environment critical for the behavior composition. None the less, the current results support the hypothesis that policy stochasticity facilitates hierarchical composition of a new behavior in both the brain and the machine.

The present study naturally translates into several important questions: how does the brain know which policies to combine under different contexts? Does the brain build dynamic models to further improve learning^25,39,40^? The selection of appropriate policies among many may require cognitive control by the prefrontal cortex, the function of which has been implicated in metalearning^20^. Model construction in deep RL provides theoretical predictions for neural representations in the brain. The comparative analysis between the two intelligent systems is imperative to explore such interesting subjects.

## Methods

### Animals

All procedures were in accordance with the Institutional Animal Care and Use Committee at Nanyang Technological University. Transgenic mice were acquired from the Jackson Laboratory (CaMKII-tTA: catalog no. 007004; TRE-GCaMP6s: catalog no. 024742). Mice were housed in a reversed light cycle (12 h:12 h) in standard cages and experiments were performed typically during the dark period. Male and female hemizygous mice were used.

### Surgery

The surgical procedure has been described previously^22^. Briefly, adult mice (aged between 7 weeks and 4 months) were anesthetized with 1–2% isoflurane and a craniotomy (~7 mm in diameter) was carried out around the bregma with a dental drill. An imaging window, constructed from a small (~6 mm in diameter) glass plug (no. 2 thickness, Thermo Fisher Scientific, catalog no. 12-540-B) attached to a larger (~8 mm in diameter) glass base (no. 1 thickness, Thermo Fisher Scientific, catalog no. 12-545-D), was placed in the craniotomy. A custom-built titanium head-plate was implanted on the window with cyanoacrylate glue and black dental acrylic (Lang Dental, catalog no. 1520BLK or 1530BLK). Buprenorphine (0.05-0.1 mg per kg of body weight), Baytril (10 mg per kg of body weight) and dexamethasone (2 mg per kg of body weight) were subcutaneously injected.

### Behavior

Mice were water restricted for ~2 weeks starting at least 3 d after the surgery. After a few days of habituation to the task apparatus, they were trained to perform two subtasks—the object manipulation task (‘Task 1’) and the licking task (‘Task 2’)—followed by the composite task. The trial structure in each task was controlled by Bpod (Sanworks) using customized codes written in MATLAB and task variables were measured by Wavesurfer (Janelia Research Campus) at the sampling rate of 2,000 Hz.

#### Task 1

Task 1 has been described previously^22^. Briefly, mice manipulated, with their right forepaws, a joystick to remotely move an object to a reward zone (4 × 4 cm^2^) located in the center of the arena (10 × 10 cm^2^). The object in the arena was a three-dimensional-printed cube attached to a 525-nm LED (Thorlabs, catalog no. LED525E) and the reward zone was indicated by another 525-nm LED. The object was moved by the joystick controlled with Arduino Leonardo (Arduino) and a motor shield (Adafruit, catalog no. 1438).

In each trial, the LEDs on the object and target were turned on. When the object reached the reward zone, it became stationary, a water (8 μl) reward was provided and the LEDs were turned off. This was followed by 4 s of a reward consumption period and 2 s of an intertrial interval. The object was reinitialized to a random position outside the reward zone after each successful trial. Each trial lasted up to 5 min and in each session mice performed up to 60 trials over ~1 h. Naive sessions were the first few sessions when another group of mice was directly introduced to the environment with the reward zone of 4 × 4 cm^2^. Expert sessions were those following completion of 60 trials for at least 2 consecutive sessions within 30 min.

In the experiment with the altered-reward function (two rewards), the reward size was changed on the left (or top) and right (or bottom) side of the reward zone (10 µl and 1 µl of water for the high- and low-reward side, respectively), but otherwise followed the same task structure as Task 1, as described previously^22^. An independent group of four and two mice was used in the environment where the reward was high on the right and bottom side of the reward zone, respectively.

To force mice to employ a more deterministic policy, a separate set of mice was trained in a modified version of the task, where the initial position was fixed (7 cm from the bottom of the arena on the left edge) in every trial.

#### Task 2

Once mice became expert at Task 1, Tasks 1 and 2 were interleaved for two sessions each to ensure that mice were able to switch between these subtasks. This was then followed by three consecutive sessions of Task 2. In Task 2, mice were trained to lick a water spout on the LED-attached, nonmovable object located in front of them. The trial started with onset of the LED, whereas the target LED used in Task 1 remained off throughout the session. Mice received a water (8 µl) reward when they successfully licked the water spout during the response period, which started 2 s after the trial onset. The trial structure was otherwise the same as Task 1. Lick events were detected with a customized touch sensor. Naive and expert sessions were defined as the first and fifth sessions, respectively.

#### Composite task

Once mice became expert at Tasks 1 and 2, they were introduced to the composite task, in which they had to combine knowledge acquired from the subtasks. In the composite task, the water spout was attached to the object that could be moved with the joystick. The trial started with onset of the object LED and the other LED used in Task 1 remained off. When the object reached the target region (5 × 0.16 mm^2^ in *x* and *y* at the bottom middle region of the arena), it became stationary and mice had to lick the water spout to trigger water dispensation (8 µl). As in the case of Task 1, after each trial the object was reinitialized to a random position outside the target region. The trial structure was the same as Task 1 and mice were trained over a total of five sessions. As a control, mice without any prior experience in Tasks 1 and 2 were trained in the composite task over the same number of sessions. In the composite task, the first session that contained more than 20 correct trials out of 60 total trials was considered as the early session for each mouse (first session for 4 mice and second session for 3 mice), whereas the fifth session was considered as the late session.

### Two-photon calcium imaging

Images were acquired using a 2p-RAM (Thorlabs) controlled with ScanImage (Vidrio Technologies) and a laser (InSight X3, Spectra-Physics), as described previously^22^. The excitation wavelength of the laser was tuned to 940 nm with a power of ~40 mW at the objective lens. The frame rate was ~5.67 Hz and the imaging resolution was 1 × 0.4 pixel per µm with 2 fields of view of 0.5 × 5 mm^2^ at the depth of ~200–300 µm (layer 2/3).

### Behavior analysis of mice

#### State-value function

As described previously^22^, the state-value function *V*(*s*) was defined as the value of each state *s*, which corresponded to the mean discounted time steps for each spatial bin and calculated according to equation (3). Mice received a reward of 1 in successful trials. The state value of the reward zone was set to be 1.

#### Action-value function

The action-value function *Q*(*s*,*a*) was defined as the value of each action *a* in a given state *s*, and described according to equation (1). *a* corresponded to one of eight discretized directions (0°, 45°, 90°, 135°, 180°, 225°, 270° and 315°) or no movement in Task 1, and lick or no lick action in Task 2. In the composite task, both types of action were considered. *V*(*s*_*t*+1_) was the value of a neighboring state in the 10 × 10 binned arena in the case of eight direction movements or the value of the same state if the movement hit the edges of the arena or in the case of no movement.

#### Policy

In Task 1 and the composite task, policy *π* was considered as a probability distribution of the object movement direction. For simplicity, *π* was displayed as a vector field with unit vectors showing the preferred action in each state and calculated by the vectorial sum of all velocity vectors in each spatial bin, obtained based on the angle and speed of the object movement over 100 ms.

Policies across the tasks and learning stages were compared with cosine similarity, where, in each spatial bin, two vectors obtained from the vectorial sum of all velocity vectors in two given conditions were multiplied and normalized by the product of their lengths. The resulting vectors were averaged across spatial bins and across sessions per animal.

#### Policy entropy

Policy entropy was computed in Task 1 in each spatial bin (*i,j*) of the 10 × 10 arena as:

6 $${{{\mathrm{Policy}}}}\,{{{\mathrm{entropy}}}}\left( {i,j} \right) = - \mathop {\sum}\limits_{{\mathrm{direction}}}^8 {\pi _{i,j}\log \pi _{i,j}}$$

where *π* is the action probability distribution computed as the normalized sum of speed of individual object movements in each direction so that the values across directions summed to 1. The mean of the policy entropy across spatial bins was then computed to determine a total policy entropy in each session.

#### State occupancy

State occupancy was determined in each session as the probability of the object residing in each of the 10 × 10 spatial bins. In the altered-reward function (two-reward) experiment, ratios of state occupancy between a high-reward side and a low-reward side were computed and averaged across sessions and across mice. The value was then compared with ratios of state occupancy obtained by randomly sampling mice with replacement 1,000× from the experiment with the original reward function.

### Imaging data analysis

Suite2p (https://github.com/cortex-lab/Suite2P) was used to perform image registration, semiautomated cell detection and neuropil correction to obtain deconvoluted calcium traces from individual cells^41^. Only those neurons with activity that passed a threshold of 20 at least once during each session were further analyzed (Task 1: 12,232, 15,324, 15,792 and 51,845 neurons for naive, expert, expert deterministic and expert altered reward function, respectively; Task 2: 3,619 and 2,563 neurons for naive and expert, respectively; composite task: 3,364 and 4,873 neurons for early and late, respectively).

Parcellation of the cortical areas was performed with the Allen Mouse Common Coordinate Framework. Each neuron was categorized to one of the five cortical regions based on the distance from the bregma. Neurons that were located at the border of the cortical areas were not classified (Task 1: M1: 938 and 3970; M2: 2,270 and 2,520; S1: 618 and 992; RSC: 1,480 and 4,481; PPC: 218 and 410 neurons for naive and expert, respectively; Task 2: M1: 815 and 862; M2: 616 and 545; S1: 374 and 250; RSC: 1,160 and 604; PPC: 51 and 19 neurons for naive and expert, respectively; composite task: M1: 904 and 1,157; M2: 659 and 912; S1: 467 and 472; RSC: 765 and 1,555; PPC: 78 and 299 neurons for early and late, respectively).

The same neurons from different sessions were identified and registered using ROIMatchPub (https://github.com/ransona/ROIMatchPub). This package performs translational shift and rotation of images to match with a reference image after manually identifying landmarks. An overlap threshold was set as 0.01 and the results were manually validated.

#### GLM

As described previously^22^, an encoding model of experimentally designed task variables was built for each neuron independently with the GLM^42–44^. The task variables included: Task 1: trial-onset and -offset times, object velocity, object position, joystick velocity and reward-onset times; Task 2: trial-onset and -offset times, joystick velocity, lick-onset times and reward-onset times; composite task: trial-onset and -offset times, object velocity, object position, joystick velocity, lick-onset times and reward-onset times. As the task variables were measured at a higher temporal sampling rate (2,000 Hz) than the imaging (5.67 Hz), they were downsampled by averaging during each imaging frame to match the imaging sampling rate.

A design matrix for GLM was constructed by representing the trial-onset and -offset times, lick-onset times and reward-onset times as boxcar functions where a value of 1 was assigned to these times and 0 elsewhere. The angle of the object velocity and joystick velocity was discretized to eight equally spaced bins (0°, 45°, 90°, 135°, 180°, 225°, 270° and 315°) to generate eight time-series data with amplitude of movement. The object position was calculated by binning the arena into 10 × 10 spatial bins. Each of the task variables was convoluted with a set of behaviorally appropriate spatial or temporal basis functions to produce task predictors (trial-onset and -offset times and lick-onset times: six evenly spaced raised cosine functions extended 2 s forward and backward in time; object and joystick velocity: six evenly spaced raised cosine functions extended 2 s forward and backward in time for each direction; object position: 100 (10 × 10) evenly spaced raised cosine functions along the two axes of the arena; reward-onset times: nine evenly spaced raised cosine functions extended 4 s forward and 2 s backward in time). GLM fitting and extraction of GLM-derived response profiles for each task variable were then performed as described previously^22^.

To examine relationships between actions (object movement for Task 1 and lick frequency for Task 2) and their neural representations, session-by-session correlations were computed across the learning stages between the traveled distance of the object and the fraction of object velocity neurons for Task 1 and between the lick frequency and the fraction of lick-onset neurons for Task 2. To further determine whether the increased fraction of the object velocity neurons was due to the increase in the object movement or learning, each session was split in half to reduce the object movement by approximately half and the fraction of the object velocity neurons was determined.

#### Analysis of *Q* representation in the mouse cortex

To determine neural representations of the *Q*_Task1_ function, *Q*_Task1_(*s*,*a*), conjunctive cells encoding the object’s spatial position and direction, which respectively corresponded to *s* and *a*, were considered. Two types of neural representations of the *Q*_Task1_ function were identified. The first class represented a high expected value of *Q*_Task1_(*s*,*a*) over actions, which was considered equivalent to *V*_Task1_(*s*). Space tuning of each conjunctive neuron was compared with *V*_*Task*1_(*s*) using Pearson’s correlation coefficient. *P* values were obtained by shuffling the object position 1,000×, computing shuffled space tuning in each neuron and calculating Pearson’s correlation coefficient between the shuffled space tuning and *V*_Task1_(*s*). The other class of neurons represented the *Q*_Task1_ function, in which the direction tuning of each neuron correlated with the *Q*_Task1_ function over eight directions with no movement in spatial bins corresponding to the top 5% of activity in the space-tuning map of the same neuron. The *Q*_*Task*1_ function was obtained by averaging *Q*_Task1_ over the spatial bins after weighting *Q*_Task1_ by the normalized activity in the same bins. To consider both direction and magnitude, Pearson’s correlation coefficient and dot product were then calculated between the direction tuning of each neuron and the *Q*_Task1_ function. *P* values were obtained by shuffling the object movement direction 1,000×, computing shuffled direction tuning in each neuron and obtaining the same metric between the shuffled direction tuning and the *Q*_Task1_ function.

To obtain a chance level of the fraction of neurons encoding *Q*_Task1_, given the same fractions of state (space)- and action (direction)-encoding neurons, state-representing neurons and action-representing neurons were randomly sampled and it was examined whether they showed conjunctive coding and whether they represented the *Q*_Task1_.

Enrichment of *Q*_Task1_-representing neurons in the original and altered-reward function (two-reward) experiments was determined by investigating the peak location of space tuning in each neuron. The fraction of neuron enriched on the high-reward side was then determined for the altered-reward function experiment in each mouse and the value was averaged across mice. For a statistical analysis, the same metric was computed by randomly sampling mice with replacement 1,000× from the experiment with the original reward function and the probability distribution was obtained.

To identify neural representations of the *Q*_Task2_ function, *Q*_Task2_(*s*,*a*), cells encoding the licking action were considered. As there was only one state in Task 2, the space tuning of neurons was not determined. A neuron was deemed to represent the *Q*_Task2_ function when the lick tuning of the neuron correlated with the *Q*_Task2_ function using Pearson’s correlation coefficient and dot product. *P* values were obtained by shuffling the lick-onset events 1,000×, computing shuffled lick tuning in each neuron and calculating the same metric between the shuffled lick tuning and the *Q*_Task2_ function. The fraction of *Q*_Task2_-encoding neurons that can be obtained by chance was determined by shuffling time-series of lick events. To examine a relationship between lick frequency and representations of *Q*_Task2_, session-by-session correlations over learning between the lick frequency and the fraction of *Q*_Task2_ function-encoding neurons were determined.

Neural representations of the *Q*_Composite_ function, *Q*_Composite_(*s*,*a*), were determined for object movement and licking in the same way as above, except that conjunctive cells encoding space and lick events for lick-related *Q* function (*Q*_Lick_ function) were considered. The *Q*_Lick_ function was obtained by averaging *Q*_Lick_ over spatial bins corresponding to the top 5% of activity in the space-tuning map of the same neuron. *Q*_Lick_ was weighted by the normalized activity in each spatial bin. A chance level of the fraction of neurons encoding *Q*_Task1_ at the early stage of the composite task was computed in the same way as Task 1. For all tasks, neurons with *P* value <0.05 with the FDR in at least one metric were considered to be *Q*_Task1_-, *Q*_Task2_- or *Q*_Composite_-representing cells.

To estimate the variability of neural representations of the *Q* functions in a given cortical area, neurons were sampled with replacement and fractions of *Q*-function-related neurons among those that are task related were computed. This procedure was repeated 1,000×.

Relationships in the fractions of *Q*_Subtask_ neurons across cortical regions between the subtasks and composite task were determined by sampling neurons with replacement 1,000× in each task independently and *P* values were computed to test whether they were positively correlated.

Neural representations of the mean *Q*_Composite_ function over actions were identified by comparing the space tuning of individual neurons and spatial map of the function with Pearson’s correlation coefficient. *P* values were then obtained by shuffling the object position 1,000× as described above. The *t*-distributed stochastic neighbor embedding (tSNE) was performed on space-tuned neurons for visualization. To test whether the mixed representations of the *Q*_Composite_ functions in the space domain of the mouse cortex were not observed by chance, the fraction of neurons correlated with the *Q*_Composite_ function averaged over actions was computed under the assumption that ‘place fields’ were uniformly distributed in the arena with the spatial basis function used in GLM^22^.

Moment-by-moment activity of *Q*_Composite_ function-encoding neurons in the mouse cortex was derived from the GLM by marginalizing out task predictors other than the object position. The activity was then thresholded at the *z*-score of 3 of the activity time-series for each neuron and sampled every 20 imaging frames for display.

#### Manifolds

Intrinsic manifolds for population neural activity were obtained by principal component analysis (PCA) to reduce dimensionality of activity of the same set of neurons between the subtasks and composite task. Activity during each trial and preceding 2 s was concatenated within each session. The coefficient obtained by PCA from the composite task was applied to population activity from the subtask to embed it in the same PC space. The resulting manifolds derived from the subtasks and composite task were compared using the KL divergence estimation^29^, which detects whether two sets of data samples were drawn from the same distribution. The KL divergence between the two manifolds was therefore inversely related to their overlaps in the PC space. The KL divergence was computed using the scipy.spatial.cKDTree module in the SciPy library in Python. Briefly, the Euclidean distance was calculated between a given sample of the subtask manifold data in the PC space and its nearest neighbor within the same data (randomly sampled *n* points). Similarly, the Euclidean distance was also determined between the same sample of the subtask manifold data and its nearest neighbor in the sampled manifold data of the composite task. The KL divergence was then estimated between the two resulting vectors (**r** and **s**) containing *n* elements according to:

7 $${{{\mathrm{KL}}}}\,{{{\mathrm{divergence}}}} = - \mathop {\sum}\limits_{i = 1}^n {\log \left( {\frac{{\mathbf{r}}}{{\mathbf{s}}}} \right) \times \frac{d}{n} + \log \left( {\frac{m}{{n - 1}}} \right)}$$

where *d* is the number of dimensions and *m* the number of samples randomly obtained from the subtask manifold data; *n* (1,000 for Task 1 and 500 for Task 2) was always the same as *m*.

To compute *P* values, the neuron index for the composite task was randomly shuffled before computing PCA and population neural activity obtained from the subtasks was embedded in this PC space. KL divergence was then computed between the resulting distributions.

#### Object movement direction selectivity

To determine the broadness of object direction tuning of each neuron, the selectivity index (SI) was determined according to:

8 $${{{\mathrm{Selectivity}}}}\,{{{\mathrm{index}}}} = 1 - {{{\mathrm{Circular}}}}\,{{{\mathrm{variance}}}}$$

The circular variance was calculated according to:

9 $${{{\mathrm{Circular}}}}\,{{{\mathrm{variance}}}} = 1 - \left\| {\frac{{\mathop {\sum}\nolimits_k {r_ke^{i2\theta _k}} }}{{\mathop {\sum}\nolimits_k {r_k} }}} \right\|$$

where *k* is the direction index, *r*_*k*_ the neural activity in response to the *k*th direction and *θ* an angle expressed in radians^45^.

### Deep reinforcement learning

#### Environment

A customized OpenAI’s gym environment was created with continuous state and action spaces to simulate the behavior paradigm designed for mice. The arena was 2.0 × 2.0 arbitrary units (a.u.) in size. The states were agent’s position in *x* and *y* coordinates and velocity. The movement variable ranged from −0.1 to 0.1 in *x* and *y* directions and the lick variable ranged from 0 to 0.1. If the lick variable exceeded 0.08, this was considered as a lick event. In Task 1, a reward of 1 was given when agents reached the reward zone (0.8 × 0.8 a.u.) located in the center of the arena from a random position outside the reward zone and no reward was given elsewhere. In Task 2, a reward of 1 was given only when the agent’s lick event was detected. In the composite task, a reward of 1 was given when the agents reached a target area located at the bottom center of the arena (0.1 × 0.0031 a.u.) and took a lick action. Agents became nonmovable once they reached the target area. No reward was given elsewhere. As was the case for Task 1, agents were reinitialized to a random position outside the target region at the start of each trial.

#### SAC algorithm

The SAC algorithm^23,24^ is an off-policy actor-critic algorithm used in continuous state and action spaces for a Markov decision process. The SAC algorithm considers the following objective:

10 $$J\left( \pi \right) = \mathop {\sum}\limits_{t = 0}^T {{\Bbb E}_{\left( {s_t,a_t} \right) \sim \rho _\pi }\left[ {r\left( {s_t,a_t} \right) + \alpha {{{\mathcal{H}}}}\left( {\pi \left( { \cdot |s_t} \right)} \right)} \right]}$$

where *π* is a policy, *T* the end of an episode, ${\Bbb E}$ expectation, *ρ*_*π*_ a state-action marginal of the trajectory distribution determined by *π*, *r*(*s*_*t*_,*a*_*t*_) a reward in a state *s*_*t*_ and action *a*_*t*_ at time *t*, *α* a temperature parameter to determine the relative contribution of the entropy term against the reward and ${{{\mathcal{H}}}}$ an entropy of *π*, defined as:

11 $${{{\mathcal{H}}}}\left( {\pi \left( { \cdot |s_t} \right)} \right) = - {\int} {\pi \left( { \cdot |s_t} \right)} \log \pi \left( { \cdot |s_t} \right)$$

The conventional objective of RL can be recovered when *α* becomes 0.

The SAC algorithm used in the present study was based on the OpenAI Spinning Up packages (https://spinningup.openai.com/en/latest/#). The agent was composed of ANNs with multilayered perceptrons for the actor computing *π* and critic computing the *Q* function (3 hidden layers and 256 neurons in each layer with ReLU as an activation function). Two parameterized *Q* networks were independently trained and the minimum of the two was used to mitigate positive bias in the policy improvement step. Adam was used as an optimizer for the actor and critic networks. Hyperparameters of the algorithms were: steps per epoch: 300, epoch number: 200, replay size: 1,000,000, gamma: 0.95, polyak averaging parameter: 0.995, learning rate: 0.0001, *α*: 0.02 for the stochastic policy in Tasks 1 and 2; 0 for the deterministic policy in Task 1; 0.005 for the composite task; maximum episode length: 300. A total of six agents were trained with different seeds.

In the composite task, the initial *Q*_Composite_ function was obtained either by averaging the *Q*_Subtask_ functions or by taking the maximum of the *Q*_Subtask_ functions using learned parameters of the *Q*_Subtask_ networks. As a control, the agent with a randomly initialized *Q*_Composite_ function was also trained. The early and late learning stages of the composite task were defined as the 20th and 200th out of 200 epochs, respectively.

For control initialization in the composite task learning, Task 2 was modified so that the state of the agent was located at the top middle region of the arena. This ensured that the value of the mean *Q*_Composite_ function over states and actions remained comparable with the original value before the deep RL agents were introduced to the composite task.

#### MBPO

The MBPO algorithm^25^ was based on the model-based reinforcement learning library (MBRL-Lib)^46^. MBPO constructs an ensemble of forward dynamic models using probabilistic neural networks and uses model-free SAC as a policy optimization algorithm under the learned model. MBPO uses an iterative process of data collection under the updated policy and training of new dynamic models with these data. For the model construction, the ensemble of 10 forward dynamic models was used, each of which was composed of a multilayer perceptron with 4 hidden layers and 256 units with the Sigmoid Linear Unit function as an activation function. Hyperparameters of the algorithm were selected as: model learning rate: 0.0001; model weight decay: 2 × 10^−6^; model batch size: 256; validation ratio: 0.2; frequency of model training: 200; effective model rollouts per step: 5; rollout schedule: [1,15,1,1]; number of SAC updates per step: 20; SAC updates every step: 1; and number of epochs to retain in SAC buffer: 50. The model learning rate was set to 0 when the dynamic models were not constructed. SAC hyperparameters were the same as above. A total of ten agents was trained with different seeds for each group (model and no-model groups).

### Behavior analysis of deep RL agents

The behavior of deep RL agents was obtained from the initial state by iteratively inputting new states based on previous actions defined by the actor whereas parameters for the *π* and *Q* neural networks were fixed. In each trial, this procedure was repeated until agents obtained a reward (hit) or 300 time steps elapsed (miss). A total of 3,000 episodes was performed. The state-value function, *V*(*s*), action-value function, *Q*(*s*,*a*), policy, *π* and policy entropy were computed similarly to those in mice with *γ* set to be 0.95. As in the case of mice, cosine similarity was computed to compare policies across tasks and learning stages.

#### Maximum entropy policies and composability of a new policy

To study whether maximum entropy policies were critical for behavior composition, visitation of the reward zone of the composite task was measured in Task 1 while the initial position was fixed (*x*: −0.2; *y*: −0.98). A relationship between the visitation and the return obtained during the composite task was then determined. Furthermore, to investigate the condition under which maximum entropy policies may become detrimental, deep RL agents were trained in the environment where the target regions were made identical between Task 1 and the composite task.

### Activity analysis of the ANN of deep RL agents

Space tuning of neurons in the hidden layers of the *Q* networks was determined by feeding 100,000 uniformly distributed random state and action inputs and measuring outputs of each layer after the activation function. The arena was divided into 40 × 40 spatial bins and the activity corresponding to each bin was averaged. This analysis ensured that activity was captured in the states that agents might not visit due to the limited number of trials, so that space tuning across different learning stages could be compared. Direction tuning of neurons in the hidden layers of the *Q* networks was determined by averaging neural activity corresponding to each binned direction of agent’s movement.

#### Analysis of *Q* representation in deep RL agents

Neural representations of the *Q* functions in the hidden layers of the *Q* networks and their chance-level fractions were determined in the same manner as those in mice. *Q* manifolds were also identified similarly to mice for the middle layer and additional comparisons between the naive and expert stages of the subtasks were performed.

Moment-by-moment activity of neurons encoding the *Q*_Composite_ function in deep RL agents was obtained by concatenating activity across epochs for each neuron, which was then *z*-scored and sampled every 100 steps for display.

#### Ablation in the ANN of deep RL agents

To investigate how inactivation of *Q*_Subtask_-representing neurons affects an agent’s learning in the composite task, activity of either 50% or 100% (full) *Q*_Subtask_-representing neurons in the hidden layers of the *Q* networks were set as 0. As a control, the same number of non-*Q*_Subtask_-representing neurons as the full ablation were also inactivated.

#### Distribution of *Q* representation in deep RL agents and mice

It was determined whether the *Q*_Move_ and *Q*_Lick_ functions were represented in a dedicated or distributed manner. For the ANN of deep RL agents, relative fractions of *Q*_Move_ and *Q*_Lick_ representations were obtained in the Task 1 and Task 2 subnetworks according to:

12 $$\frac{{{\mathrm{Fraction}}\,{\mathrm{in}}\,{\mathrm{Task}}\,1\left( {{\mathrm{or}}\,{\mathrm{Task}}\,2} \right){\mathrm{network}} - {\mathrm{Fraction}}\,{\mathrm{in}}\,{\mathrm{Task}}\,2 ({\mathrm{or}}\,{\mathrm{Task}}\,1) {\mathrm{network}}}}{{{\mathrm{Fraction}}\,{\mathrm{in}}\,{\mathrm{Task}}\,1\left( {{\mathrm{or}}\,{\mathrm{Task}}\,2} \right){\mathrm{network}} + {\mathrm{Fraction}}\,{\mathrm{in}}\,{\mathrm{Task}}\,2({\mathrm{or}}\,{\mathrm{Task}}\,1){\mathrm{network}}}}$$

For mice, Pearson’s correlation coefficients in the cortical distribution of *Q*_Move_ and *Q*_Lick_ representations were computed between the subtask expert and composite task early or composite task late stages.

### Statistics

For statistically significant results, *P* values were adjusted after respective correction methods for multiple comparisons, which are described in each figure legend.

### Reporting summary

Further information on research design is available in the Nature Portfolio Reporting Summary linked to this article.

## Online content

Any methods, additional references, Nature Portfolio reporting summaries, source data, extended data, supplementary information, acknowledgements, peer review information; details of author contributions and competing interests; and statements of data and code availability are available at 10.1038/s41593-022-01211-5.

## Supplementary information

Reporting Summary

Supplementary Video 1 Behavior and neural activity in Q networks of the deep RL agent at the early learning stage of the composite task. The blue dot is the current state and the red dot the lick action of the agent.

Supplementary Video 2 Behavior and neural activity in Q networks of the deep RL agent at the late learning stage of the composite task. The same visualization is used as in Supplementary Video 1.

## Data Availability

Data are available at 10.5281/zenodo.7283276 on Zenodo.

## Code availability

Code for data analysis is available at https://github.com/HiroshiMakinoLaboratory/Makino2022NatureNeuroscience on GitHub.
