# Supplementary material for: Arithmetic value representation for hierarchical behavior composition
Source: Nat Neurosci. 2022 Dec 22;26(1):140–9. doi: 10.1038/s41593-022-01211-5 (PMC9829535; doi:10.1038/s41593-022-01211-5)
Supplement: Supplementary file 1 — Reporting Summary [file 41593_2022_1211_MOESM1_ESM.pdf]

nature portfolio

Corresponding author(s): Hiroshi Makino

Last updated by author(s): Nov 22, 2022

Reporting Summary

Nature Portfolio wishes to improve the reproducibility of the work that we publish. This form provides structure for consistency and transparency in reporting. For further information on Nature Portfolio policies, see our Editorial Policies and the Editorial Policy Checklist.

Statistics

For all statistical analyses, confirm that the following items are present in the figure legend, table legend, main text, or Methods section.

n/a	Confirmed	
☐	☒	The exact sample size (n) for each experimental group/condition, given as a discrete number and unit of measurement
☐	☒	A statement on whether measurements were taken from distinct samples or whether the same sample was measured repeatedly
☐	☒	The statistical test(s) used AND whether they are one- or two-sided<br>*Only common tests should be described solely by name; describe more complex techniques in the Methods section.*
☒	☐	A description of all covariates tested
☐	☒	A description of any assumptions or corrections, such as tests of normality and adjustment for multiple comparisons
☐	☒	A full description of the statistical parameters including central tendency (e.g. means) or other basic estimates (e.g. regression coefficient) AND variation (e.g. standard deviation) or associated estimates of uncertainty (e.g. confidence intervals)
☐	☒	For null hypothesis testing, the test statistic (e.g. F, t, r) with confidence intervals, effect sizes, degrees of freedom and P value noted<br>*Give P values as exact values whenever suitable.*
☒	☐	For Bayesian analysis, information on the choice of priors and Markov chain Monte Carlo settings
☒	☐	For hierarchical and complex designs, identification of the appropriate level for tests and full reporting of outcomes
☐	☒	Estimates of effect sizes (e.g. Cohen's d, Pearson's r), indicating how they were calculated

Our web collection on statistics for biologists contains articles on many of the points above.

Software and code

Policy information about availability of computer code

Data collection	Behavior data were acquired with Bpod (Sanworks) and WaveSurfer 0.965 (Janelia Research Campus). Calcium imaging data were acquired with a two-photon random access mesoscope (Thorlabs) controlled by ScanImage 2017 (Vidrio Technologies).
Data analysis	Imaging data were pre-processed with Suite2p. The same neurons from different sessions were identified with ROIMatchPub. Parcellation of the cortical areas was performed with the Allen Mouse Common Coordinate Framework. Behavior and imaging data were analyzed with custom codes written in MATLAB and Python. For deep RL, custom OpenAI gym environments were created and agents were trained with the soft actor-critic algorithm based on the OpenAI Spinning Up package or the model-based policy optimization algorithm based on the model-based reinforcement learning library (MBRL-Lib). Deep RL results were analyzed with custom codes written in MATLAB and Python. Code for data analysis is available at https://github.com/HiroshiMakinoLaboratory/Makino2022NatureNeuroscience on GitHub.

For manuscripts utilizing custom algorithms or software that are central to the research but not yet described in published literature, software must be made available to editors and reviewers. We strongly encourage code deposition in a community repository (e.g. GitHub). See the Nature Portfolio guidelines for submitting code & software for further information.

Data

Policy information about availability of data

All manuscripts must include a data availability statement. This statement should provide the following information, where applicable:

- Accession codes, unique identifiers, or web links for publicly available datasets
- A description of any restrictions on data availability
- For clinical datasets or third party data, please ensure that the statement adheres to our policy

Data are available at https://doi.org/10.5281/zenodo.7283276 on Zenodo.

Human research participants

Policy information about studies involving human research participants and Sex and Gender in Research.

Reporting on sex and gender	*Use the terms sex (biological attribute) and gender (shaped by social and cultural circumstances) carefully in order to avoid confusing both terms. Indicate if findings apply to only one sex or gender; describe whether sex and gender were considered in study design whether sex and/or gender was determined based on self-reporting or assigned and methods used. Provide in the source data disaggregated sex and gender data where this information has been collected, and consent has been obtained for sharing of individual-level data; provide overall numbers in this Reporting Summary. Please state if this information has not been collected. Report sex- and gender-based analyses where performed, justify reasons for lack of sex- and gender-based analysis.*
Population characteristics	*Describe the covariate-relevant population characteristics of the human research participants (e.g. age, genotypic information, past and current diagnosis and treatment categories). If you filled out the behavioural & social sciences study design questions and have nothing to add here, write "See above."*
Recruitment	*Describe how participants were recruited. Outline any potential self-selection bias or other biases that may be present and how these are likely to impact results.*
Ethics oversight	*Identify the organization(s) that approved the study protocol.*

Note that full information on the approval of the study protocol must also be provided in the manuscript.

Field-specific reporting

Please select the one below that is the best fit for your research. If you are not sure, read the appropriate sections before making your selection.

☒ Life sciences ☐ Behavioural & social sciences ☐ Ecological, evolutionary & environmental sciences

For a reference copy of the document with all sections, see nature.com/documents/nr-reporting-summary-flat.pdf

Life sciences study design

All studies must disclose on these points even when the disclosure is negative.

Sample size	Sample sizes were determined according to those reported in previous publications (Makino et al. 2017, Suhaimi et al. 2022).
Data exclusions	Animals were excluded when the optical window was occluded for imaging. The criterion for the exclusion was pre-determined.
Replication	All results were replicated across multiple animals and deep RL agents (n >= 6) per group.
Randomization	Animals were randomly assigned to each experimental group.
Blinding	Blinding was not performed during group allocation but data analysis was conducted regardless of the identity or experimental group of animals.

Reporting for specific materials, systems and methods

We require information from authors about some types of materials, experimental systems and methods used in many studies. Here, indicate whether each material, system or method listed is relevant to your study. If you are not sure if a list item applies to your research, read the appropriate section before selecting a response.

Materials & experimental systems

n/a	Involved in the study
☒	☐ Antibodies
☒	☐ Eukaryotic cell lines
☒	☐ Palaeontology and archaeology
☐	☒ Animals and other organisms
☒	☐ Clinical data
☒	☐ Dual use research of concern

Methods

n/a	Involved in the study
☒	☐ ChIP-seq
☒	☐ Flow cytometry
☒	☐ MRI-based neuroimaging

Animals and other research organisms

Policy information about studies involving animals; ARRIVE guidelines recommended for reporting animal research, and Sex and Gender in Research

Laboratory animals	Transgenic mice were acquired from the Jackson Laboratory (CaMKII-tTA: 007004; TRE-GCaMP6s: 024742). Mice were aged 2 to 4 months old when surgery was performed. Mice were housed in a reversed light cycle (12h:12h) in standard cages at around 21 °C and 62% humidity. Experiments were typically performed during the dark phase.
Wild animals	No wild animals were used.
Reporting on sex	Both male and female mice were used and randomly assigned to each experimental group.
Field-collected samples	No field-collected samples were used.
Ethics oversight	All procedures were in accordance with the Institutional Animal Care and Use Committee at Nanyang Technological University.

Note that full information on the approval of the study protocol must also be provided in the manuscript.
